# The Crosstalk between Autophagy and Nrf2 Signaling in Cancer: from Biology to Clinical Applications

**DOI:** 10.7150/ijbs.103187

**Published:** 2024-11-11

**Authors:** Chan Shan, Yuan Wang, Yin Wang

**Affiliations:** 1 Key Laboratory of Maternal & Fetal Medicine of National Health Commission of China, Shandong Provincial Maternal and Child Health Care Hospital Affiliated to Qingdao University, Jinan, 250014, China.; 2 Institute of Translational Medicine, College of Medicine, Qingdao University, Qingdao 266021, China.

**Keywords:** Autophagy, Nrf2 signaling, Crosstalk, Cancer biology, Cancer therapy

## Abstract

Autophagy is a catabolic process that has been conserved throughout evolution, serving to degrade and recycle cellular components and damaged organelles. Autophagy is activated under various stress conditions, such as nutrient deprivation, viral infections, and genotoxic stress, and operates in conjunction with other stress response pathways to mitigate oxidative damage and maintain cellular homeostasis. One such pathway is the Nrf2-Keap1-ARE signaling axis, which functions as an intrinsic antioxidant defense mechanism and has been implicated in cancer chemoprevention, tumor progression, and drug resistance. Recent research has identified a link between impaired autophagy, mediated by the autophagy receptor protein p62, and the activation of the Nrf2 pathway. Specifically, p62 facilitates Keap1 degradation through selective autophagy, leading to the translocation of Nrf2 into the nucleus, where it transcriptionally activates downstream antioxidant enzyme expression, thus safeguarding cells from oxidative stress. Furthermore, Nrf2 regulates p62 transcription, so a positive feedback loop involving p62, Keap1, and Nrf2 is established, which amplifies the protective effects on cells. This paper aims to provide a comprehensive review of the roles of Nrf2 and autophagy in cancer progression, the regulatory interactions between the Nrf2 pathway and autophagy, and the potential applications of the Nrf2-autophagy signaling axis in cancer therapy.

## 1. Introduction

Autophagy is a cellular process in which eukaryotic cells degrade cytoplasmic proteins and damaged organelles through the action of lysosomes under the regulation of autophagy-related genes (ATGs) 1. This process serves to prevent cell damage, promote cell survival in the absence of nutrients, and respond to cytotoxic stimuli. Autophagy plays a crucial role in both normal physiological functions and pathological conditions within the body 2. Under physiological conditions, autophagy functions to eliminate aging and damaged biological macromolecules and organelles, thereby preserving the normal biological functions of cells. During metabolic stress states, autophagy facilitates the degradation of aging and damaged biological components, providing cells with the necessary energy sources and materials for reconstruction in harsh environments. Importantly, dysregulation of autophagy has been implicated in the pathogenesis and progression of various diseases, including autoimmune disorders, neurological conditions, cardiovascular and cerebrovascular ailments, metabolic diabetes, and notably, cancer 3. Current research indicates that autophagy plays a dual role in cancer, influenced by factors such as tumor stage, specific oncogenic mutations, and the surrounding cellular environment. Autophagy is widely recognized to be a suppressor of tumorigenesis; however, in established tumors, it facilitates the unregulated proliferation of cancer cells and their increased metabolic activity, resulting in dependence on autophagy for tumor maintenance 4, 5. Moreover, autophagy plays a crucial role not only in cancer cells but also in adjacent stromal cells and the tumor microenvironment (TME), which are intricately linked to tumor growth and drug resistance 6, 7. Therefore, comprehending the molecular mechanisms involved in autophagic control has significant potential for devising efficacious approaches for the prevention and treatment of cancer.

Nuclear factor (erythroid derived-2)-like 2 (Nrf2), a pivotal transcription factor encoded by *NFE2L2* that is responsible for regulating antioxidant stress, assumes a critical function in triggering the body's antioxidant response 8. In normal physiological conditions, the interaction between Nrf2 and Kelch-like ECH-associated protein 1 (Keap1) facilitates the ubiquitination of Nrf2, leading to its degradation by the 26S proteasome 9. However, during oxidative stress, Keap1 undergoes conformational changes that disrupt the ubiquitination process of Nrf2. Consequently, Nrf2 translocates to the nucleus, where it binds to antioxidant response elements (AREs) and initiates the transcription of various cytoprotective genes, such as NAD(P)H quinone dehydrogenase 1 (NQO1), heme oxygenase 1, superoxide dismutase, glutamate cysteine ligase (GCL), and catalase.

Over the past decade, numerous studies have provided evidence of the crosstalk between autophagy and Nrf2 signaling, which is facilitated by the autophagy adaptor p62/sequestosome 1 10-13. In this scenario, various factors, such as oxidative stress, metabolic disorders, diseases, and autophagy inhibition, trigger the transcription of p62, leading to its accumulation in the cytoplasm. The aggregation of p62 then directly interacts with Keap1, resulting in the degradation of Keap1 through a selective autophagic pathway, which ultimately leads to the continuous activation of Nrf2 and the upregulation of genes encoding antioxidant enzymes. Nuclear Nrf2 also promotes the overexpression of the *p62* gene, establishing a p62-Keap1-Nrf2-positive feedback loop. An increasing body of research has substantiated the pivotal significance of this feedback loop in the progression of cancer 12, 13. In this review, we specifically focus on the regulatory functions of autophagy and Nrf2 signaling, as well as their crosstalk, in the progression of cancer. We also discuss their potential applications in cancer therapy.

## 2. The role of autophagy in cancer

### 2.1. Molecular mechanisms of autophagy

**Figure 1:**
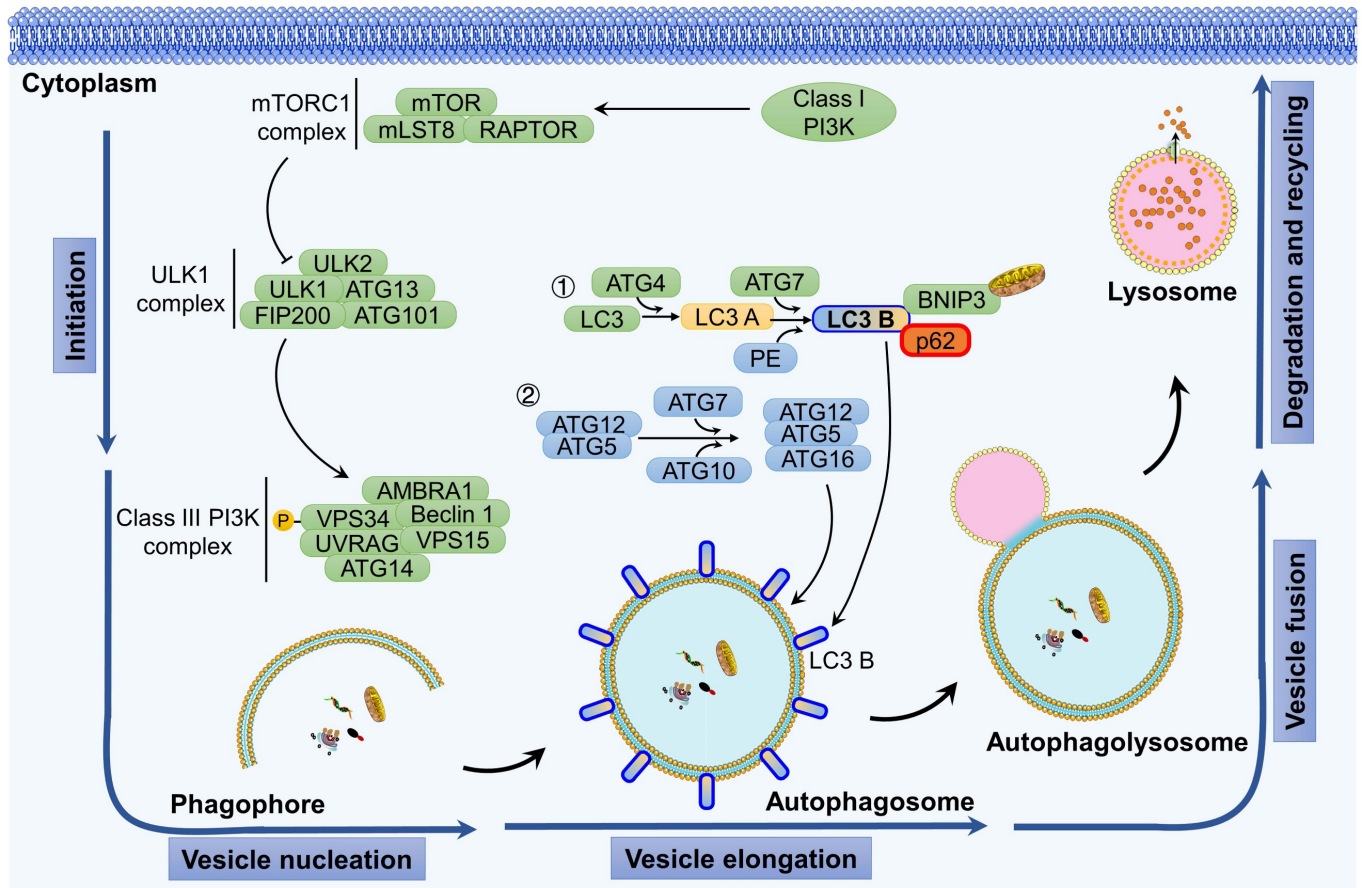
Autophagy is a cellular process that is activated in response to internal stressors, such as hypoxia or nutrient deprivation. Autophagy initiation involves activation of the class III phosphatidylinositol 3-kinase (PI3K) complex, which is composed of Beclin 1, AMBRA1, ATG14L, VPS15, and VPS34. This activation is facilitated by the ULK1 complex, which comprises FIP200, ATG13, and ATG101. Activation of the class III PI3K complex initiates vesicle nucleation, which serves as a scaffold for membrane expansion. During the membrane expansion phase, two ubiquitin-like conjugation systems play a role: the ATG5-ATG12 complex combined with ATG16L1 and LC3 combined with PE. These systems are responsible for elongating and forming autophagosomes. The final crucial step involves the fusion of autophagosomes with lysosomes to create autophagic lysosomes, where biological macromolecules are degraded and recycled.

Autophagy serves as the fundamental process for the degradation and recycling of cellular components 1. It involves the breakdown of impaired, aged, or dysfunctional organelles to promptly supply energy and essential resources for the maintenance of normal cellular activities. The autophagy process primarily consists of five distinct stages: initiation of autophagy, vesicle nucleation, vesicle elongation, fusion of autophagosomes with lysosomes, and ultimately the degradation of the enclosed contents (Figure 1).

Autophagy is triggered by both extracellular stimulation (e.g., nutrient shortage, hypoxia-ischemia, and growth factor induction) and intracellular factors (e.g., metabolic stress, organelle aging, protein misfolding, and DNA damage) 14. The initiation of autophagy is regulated by the protein complex of the target of rapamycin (TOR) kinase. Specifically, the mammalian TOR (mTOR) is activated in response to cellular stress, which, in turn, activates downstream serine/threonine UNC-51-like kinases 1 and 2 (ULK1 and ULK2). ULK1 and ULK2 then form a complex with the 200 kDa family-interacting proteins ATG101 (also known as FIP200, a mammalian homolog of ATG17) and ATG13, thereby initiating the autophagy process.

Two distinct ubiquitin-like conjugation systems are involved in the formation and maturation of autophagosomes: the ATG5-ATG12 complex conjugated with ATG16L1 and microtubule-associated protein 1 light chain 3 (MAP1LC3, also known as LC3) conjugated with lipid phosphatidylethanolamine (PE) 1. Upon activation by upstream signals, ATG12 and ATG5 undergo sequential conjugation facilitated by ATG7 and ATG10, ultimately leading to the formation of a complex with ATG16L, which plays a critical role in the assembly of autophagosomes. Following translation, pro-LC3 is immediately cleaved by ATG4 at the C-terminal peptide, exposing a glycine residue site and resulting in the formation of LC3 A, which is then distributed throughout the cytoplasm. Through the action of the E1-like enzyme ATG7, the E2-like enzyme ATG3, and the E3-like enzyme ATG12-ATG5-ATG16L, LC3 A covalently binds with PE to generate LC3-PE, which is also referred to as LC3 B. The lipophilicity of PE facilitates the binding of LC3 to the autophagosome membrane, thereby mediating membrane elongation and the selective degradation of cellular components. LC3 B has been widely used as a marker to monitor the autophagy process 15-17.

The fusion of autophagosomes and lysosomes is a critical process for cells to carry out autophagy, which involves the breakdown and recycling of cellular waste 18. Specifically, autophagosomes initially fuse with endosomes to create late endosomes and then form autolysosomes through fusion with lysosomes. The process of autophagosome-lysosome fusion begins with the merging of the outer membrane of autophagosomes with lysosomes and concludes with the degradation of the inner membrane of autophagosomes and the release of their contents into the lysosomal lumen. Several functional proteins play a role in the fusion of autophagosomes and lysosomes, including small GTPases, RAB proteins (RAB5 and RAB7), the UV radiation resistance-associated gene protein (UVRAG), and SNARE proteins (VAMP8 and STX17) 1.

Following fusion, the cellular material transported by autolysosomes undergoes degradation by hydrolases in the lysosomes, resulting in the production of substrates that can be circulated and metabolized 3. Selective autophagy exhibits distinct characteristics compared to nonselective autophagy. For instance, cargo items must possess an ubiquitination label and attach to the inner surface of the phagophore via ATG8s, thereby forming a large aggregate that facilitates phagophore formation 19.

In addition, mitophagy, the process by which damaged mitochondria are cleared, is triggered in various situations such as hypoxia, mitochondrial DNA damage, oxidative stress, and mitochondrial depolarization 20. In mammals, the classical pathways that mediate mitophagy can be categorized into two main pathways: ubiquitin dependent pathways and non-ubiquitin dependent. Among these, the PTEN-induced putative kinase 1 (PINK1)/PARKIN signaling pathway has been extensively studied 21. Under normal conditions, PINK1 enters the inner mitochondrial membrane through the TOM/TIM complex and is then cleaved by proteases in the inner membrane and subsequently degraded by proteasomes. When mitochondria are damaged, the mitochondrial membrane potential decreases, preventing PINK1 from entering the inner membrane and causing its accumulation on the outer membrane of mitochondria. Subsequently, the receptor protein for autophagy accumulates on the outer membrane of mitochondria. Mitochondria are then transported to the autophagosome with the assistance of LC3 and ultimately degraded by lysosomes.

Together, elucidating the operational mechanisms of individual stages within the autophagy process and delineating the regulation and interplay of the pivotal receptor proteins involved in this process are essential. This is important for enhancing the understanding of the quality control mechanism in autophagy and cellular homeostasis.

### 2.2. Bipolar properties of autophagy in cancer progression

The intricate involvement of autophagy in the regulation of cancer development has been the subject of extensive research. The impact of autophagy on the fate of cancer cells varies depending on factors such as the type of cancer, its stage, and the genetic background of the individual 22. On the one hand, autophagy functions as a quality control mechanism that preserves genomic stability by removing metabolic waste, damaged organelles, and other cellular components 23-25. Based on this perspective, autophagy protects cells from damage induced by various microenvironmental stimuli, inflammatory responses, and nutritional imbalances, thereby acting as a tumor suppressor in the early stages of cancer 4, 26, 27. On the other hand, as cancer progresses, cancer cells exploit autophagy as a protective and defensive mechanism, allowing cancer cells to sustain their metabolism, growth, and survival, ultimately facilitating cancer progression, metastasis, and the development of drug resistance 5, 28, 29. The dual roles of autophagy in Table 1.

#### Evidence and suppressive functions of autophagy in tumorigenesis

As previously stated, autophagy serves as a mechanism employed by organisms to maintain metabolic, protein, and organelle integrity and to eliminate impaired proteins and organelles that have accumulated during periods of stress 1. Consequently, autophagy plays a crucial role in preventing the development of tumors, especially in the early stages of tumorigenesis 4, 26, 27. A growing body of research has revealed that defective autophagy promotes tumorigenesis, typically because of the deletion or impairment of specific ATGs within a particular tumor. An illustrative example is the tumor suppressor gene *Beclin 1*, which is implicated in the initiation of autophagy in the context of cancer. Monoallelic loss of Beclin 1 is sufficient to induce the development of breast cancer, ovarian cancer, B cell lymphoma, melanoma, and other malignancies 30-32. However, the upregulation of Beclin 1 is associated with enhanced autophagic flux, a reduction in tumorigenesis, and the inhibition of malignant characteristics 33. Additionally, dysfunctions in ATGs, such as ATG12, ATG14, and ATG5, can affect the autophagy process, thereby influencing the initiation and progression of cancer, as well as response to chemotherapy. For instance, in head and neck squamous cell carcinoma (HNSCC), the depletion of ATG12 leads to a diminished autophagic flux, which subsequently inhibits the proliferation of cancer cells and enhances their susceptibility to chemotherapeutic agents 34. In the context of oral cancer, ATG5-dependent autophagy plays a crucial role in preserving tumor stemness, facilitating self-renewal, and conferring cisplatin resistance in oral CD44^+^ cells 35. Overall, the downregulation and deletion of ATGs have been shown to increase the incidence of malignancies, indicating that intact autophagy plays a critical role in tumor suppression.

The PI3K/AKT/mTORC1 signaling pathway and its associated proteins also play a crucial role in the regulation of autophagy and tumorigenesis 36. Autophagy can be inhibited by PI3K inhibitors, such as wortmannin and 3-Methylamphetamine, while rapamycin, an mTORC1 inhibitor, effectively induces autophagy. AKT also participates in the regulation of autophagy through its interaction with various proteins, including p62, Forkhead Box O3, ULK1, Phafin2, and Beclin 1. Research has demonstrated that the presence of chronic inflammation can elevate the likelihood of developing cancer, while autophagy serves as a fundamental mechanism within the inflammasome 37. Impairments in autophagy have the potential to induce tissue harm, necrosis, persistent inflammation, and genetic instability, thereby augmenting the occurrence of cancer through alterations in the TME, heightened oxidative stress, and the generation of oncogenic mutations 38. In conclusion, the a reduction in the expression of functional autophagy genes, defects in ATGs, and the inhibition of signaling pathways associated with autophagy all contribute to an elevated occurrence of cancer, suggesting that maintaining intact autophagy is crucial for its antitumor effects.

#### The role of autophagy in promoting cancer development

Preliminary evidence indicating the involvement of autophagy in cancer sustenance was observed through the detection of elevated levels of LC3 and lipidated LC3 (LC3 B) in certain tumor tissues, suggesting an accumulation of autophagosomes 39. Further investigations have demonstrated that autophagy plays a crucial role in facilitating cancer progression by eliminating harmful oxygen free radicals and damaged proteins, preserving mitochondrial function, and meeting the metabolic and survival requirements of cancer cells in stressed conditions 22. Tumor cells rely primarily on glycolysis because of metabolic alterations; however, mitochondrial function remains essential for specific anabolic processes. Autophagy plays a critical role in maintaining mitochondrial integrity, and deficiencies in autophagic processes can lead to the accumulation of dysfunctional mitochondria 40, 41.

In addition to the direct examination of cancer cells, evaluating the effects of autophagy on surrounding and distant stromal cells within the host organism is needed in the investigation of cancer autophagy. Research utilizing murine models has demonstrated that the systemic deletion of ATG7 results in widespread autophagy deficiencies across the host, which correlate with a significant regression of KRAS-driven tumors 42. Likewise, a significant regression of KRAS-driven pancreatic cancer was observed in ATG4B-mutated mouse models of systemic autophagy inhibition 43. These findings imply that autophagy, both in the host organism and within the cancer cells themselves, plays a critical role in facilitating cancer progression.

Moreover, tumors should not be regarded as isolated entities; rather, they are intricately linked and coordinated with the TME, which consists of stromal and immune cells, among others. Research has indicated that autophagic processes in stromal cells can enhance the anabolic activities of cancer cells, thereby facilitating tumor progression. For instance, pancreatic stellate cells can produce pyruvate through an autophagy-dependent mechanism, which is subsequently utilized by pancreatic cancer cells for oxidative metabolism 44. Another important instance is cancer-associated fibroblasts (CAFs), which are significant cellular components within the tumor stroma. Metabolites generated through autophagy of CAFs, including glutamine, free fatty acids, and ketones, can be utilized by cancer cells as nutritional resources 45. Autophagy in CAFs also facilitates the release of interleukin-6, interleukin-8, and a range of other cytokines, contributing to the malignant progression of HNSCC 46. Collectively, these investigations highlight the significant function of stromal cell autophagy in advancing tumors, suggesting that modulation of this process may yield novel perspectives for cancer treatment.

Autophagic degradation may also affect stromal and immune cells, facilitating tumor progression. For instance, in the context of pancreatic ductal adenocarcinoma, hypoxia-induced autophagy in pancreatic stellate cells leads to lumica degradation within the extracellular matrix, which subsequently promotes cancer progression 47. Additionally, autophagy contributes to the progression of cancer by influencing the functionality of immune cells. In liver cancer, autophagy is implicated in NF-κB p65 degradation, which drives the differentiation of bone marrow-derived macrophages into tumor-promoting M2 phenotypes via the TLR2 signaling pathway 48. Another separate study indicated that inhibition of autophagy in myeloid-derived suppressor cells disrupts lysosomal degradation functions, resulting in an increased expression of MHC-II and activation of CD4^+^ T cells, thereby enhancing antitumor immunity and inhibiting melanoma progression 49. Future investigations into the mechanisms by which autophagy influences the function of tumor stromal cells and immune cells within TME are essential, as these processes significantly affect cancer progression.

#### Pro-metastasis and anti-metastasis functions of autophagy in cancer

Metastasis is a significant contributor to cancer progression and mortality in affected individuals. The involvement of autophagy in cancer metastasis is currently a subject of debate within the scientific community. Autophagy has been implicated in facilitating cancer metastasis through various biological mechanisms, including the promotion of cancer cell migration and invasion 50, maintenance of cancer cell dormancy 51, preservation of cancer stem cells (CSCs) 52, suppression of senescence in cancer cells 53, regulation of epithelial-mesenchymal transition (EMT) 54, adaptation to nutrient deprivation and hypoxia conditions 5, 34, and cancer cell survival within the microenvironment outside the lesion, among other factors. Currently, the association between autophagy and cancer metastasis has been demonstrated across multiple cancer types. This understanding has facilitated the advancement of promising therapeutic approaches to managing cancer metastasis and recurrence through the inhibition of autophagy. For instance, fusobacterium nucleatum outer membrane vesicles (Fn OMVs) have been demonstrated to induce autophagy in oral cancer cells, decrease the expression of EMT-associated proteins, enhance migration and invasive capabilities, and facilitate lung metastasis *in vivo* 55. These findings suggests that the inhibition of autophagy induced by Fn OMVs, whether through chemical or biological interventions, may hinder the metastatic progression of oral cancer, offering novel avenues for therapeutic strategies in the management of this malignancy. Moreover, the resistance exhibited by chemotherapy agents presents a significant challenge in clinical practice. Zamora *et al.* shed light on a novel mechanism underlying paclitaxel-induced metastasis in breast cancer 56. Mechanistically, paclitaxel inhibits the migration and adhesion of lymphoendothelial cells via an autophagy-dependent pathway, simultaneously enhancing their permeability and thus facilitating metastasis and malignant progression of breast cancer to sentinel lymph nodes. Advances in innovative combination therapies may offer promising strategies to mitigate and potentially reverse paclitaxel resistance in breast cancer.

By contrast, recent research has underscored the critical role of autophagy in suppressing cancer metastasis, particularly highlighting its influence on cancer cell dormancy. In various malignancies, including breast cancer, prostate cancer, and small-cell lung cancer, cancer cells can spread from the primary tumor to secondary sites, entering a stage of growth arrest that may persist for more than a decade 51. Under certain conditions, these dormant cells can reactivate, leading to metastatic recurrence. A growing body of evidence supports the notion that autophagy plays a pivotal role in inhibiting cancer cell dormancy, particularly in the context of metastatic colonization and proliferation. For instance, the ablation of ATG3 and ATG5 in dormant breast cancer cells prompts them to exit dormancy, resulting in the emergence of a malignant phenotype similar to CSCs, which is associated with aggressive growth and metastatic recurrence 57, 58. Notably, cells that reactivated because of autophagy inhibition often exhibit genomic instability; however, the implications for cancer metastasis warrant further investigation. Additionally, as previously noted, autophagy that protects cellular integrity is implicated in metastasis primarily through the maintenance of cellular homeostasis, protecting cancer cells from death 1. Conversely, in certain contexts, autophagy may hinder metastatic processes by preventing necrosis of cancer cells and infiltration of inflammatory cells 59. Autophagic cell death can also contribute to the suppression of cancer cell metastasis by inducing the demise of these cells 60. While an increasing body of evidence supports the significant role of autophagy in cancer metastasis, the precise functions and effects of autophagy in specific microenvironments require further investigation.

## 3. Nrf2 signaling in cancer

### 3.1. The structure of Nrf2

**Figure 2:**
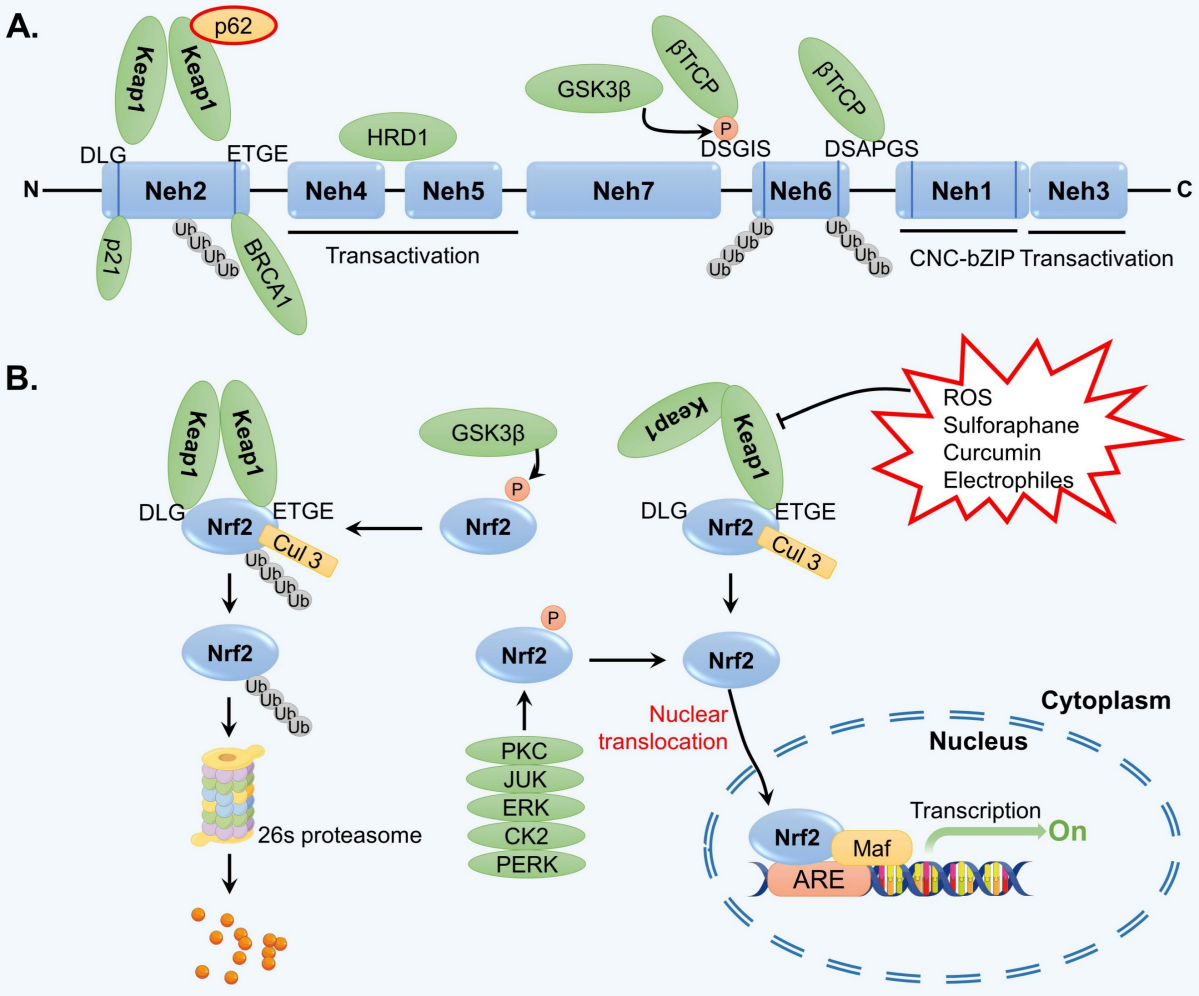
The structure of Nrf2 and regulation of Nrf2 signaling. (A) Nrf2 is comprised of seven Nehs, which play a role in mediating its interaction with adaptors. (B) Under normal physiological conditions, the Keap1-Cul3-E3 ubiquitin ligase typically targets various lysine residues in the Nrf2 Neh2 domain, located between DLG and ETGE motifs, facilitating ubiquitination that subsequently leads to degradation by the 26S proteasome. Upon exposure to reactive oxygen species (ROS) and electrophilic stress, specific cysteine residues in Keap1 undergo modifications, resulting in structural alterations in the Keap1-Cul3-E3 ubiquitin ligase complex. This disrupts the ubiquitination of Nrf2, prompting its translocation to the nucleus where it binds to AREs in target genes through heterodimerization with the MAF protein. Consequently, this initiates a cascade of gene expression that serves to protect the cell.

Throughout the course of evolution, cells have developed the capacity to withstand various internal and external pressures and stimuli. One particularly significant transcription factor, Nrf2, plays a crucial role in facilitating the regulation of the cellular antioxidant stress response 61. Structurally, Nrf2 is a protein that consists of seven highly conserved ECH homology domains (Nehs). One of these domains, known as Neh1, contains a leucine CNC-bZIP region that facilitates the binding of Nrf2 to the ARE in DNA through interaction with the muscle tendon membrane fibrosarcoma (Maf) protein in the cell nucleus 62. As a result, the transcription of various enzymes involved in antioxidant defense, ubiquitination, phase II detoxification, and proteasome activity is activated, thereby counteracting oxidative stress in cells. The Neh2 domain of Nrf2 contains ETGE and DLG domains, which exhibit high and low affinities for Keap1, respectively 63. Neh2 is responsible for the degradation of Nrf2 and negatively regulates its transcriptional activity. Neh3 can interact with the transcription coactivator chromodomain-helicase-DNA-binding protein 6 and collectively modulate the activation of ARE-dependent gene transcription 64. The Neh4 and Neh5 domains of Nrf2 can bind to the activating transcription factor cAMP response element-binding protein, facilitating the translocation of Nrf2 into the nucleus and its subsequent binding to ARE in the form of the Nrf2-Maf complex 65. Neh6, which contains numerous serine residues, is involved in the ubiquitination degradation of Nrf2 mediated by the Skp1-Cul1-Rbx1/Roc1-β-TrCP ubiquitin ligase complex and does not rely on Keap1 66. Neh7 of Nrf2 primarily functions to recognize the retinoid X receptor alpha 67. Nrf2 also possesses crucial phosphorylation sites that regulate the binding and dissociation of Nrf2 and Keap1. The structure of Nrf2 is shown in Figure 2A.

### 3.2. Nrf2 signaling

The activity of Nrf2 signaling is mainly regulated by the negative modulator Keap1. Keap1 can form homodimers and interact with Nrf2 through the ETGE and DLG sequences 68. In normal physiological conditions, the Keap1-Nrf2 complex binds to the E3 ubiquitin ligase Cullin 3 (Cul3) via the Neh6 domain of Nrf2, leading to the ubiquitination and subsequent degradation of Nrf2 by the 26S proteasome 69. However, when exposed to oxidative stress or chemical stimulation, Keap1 undergoes conformational changes and dissociates from Nrf2, interrupting the ubiquitination and degradation of Nrf2 70. Consequently, Nrf2 is released and translocated to the nucleus, where it functions as a transcription factor. Specifically, Nrf2 activates the transcription of a series of antioxidant enzymes by binding to Maf and ARE (Figure 2B).

It has been observed that Nrf2 can also be regulated independently of Keap1. Several studies have demonstrated that certain kinases, including protein kinase C, casein kinase II, protein kinase R-like endoplasmic reticulum kinase, c-Jun N-terminal kinase, and extracellular signal-regulated kinase, are capable of phosphorylating Nrf2 and facilitating its translocation into the nucleus 71-74. Conversely, the phosphorylation of Nrf2 by glycogen synthase kinase-3β and mitogen-activated protein kinase can lead to its degradation 75.

Nrf2 signaling can also be modulated by genetic and epigenetic factors. Studies have demonstrated the presence of a potential ARE sequence within the promoter region of *NFE2L2*, suggesting the existence of a plausible feedback regulatory mechanism that can modulate the transcriptional activity of Nrf2 itself 63. In addition, some transcription factors, such as AhR, have been demonstrated to modulate the transcriptional activity of Nrf2 by altering the TME and promoting redox homeostasis in breast cancer 76, 77. The modulation of *NFE2L2* by transcription factors plays a significant role in metabolic reprogramming and the malignant progression of cancer. In the context of HNSCC, C-MYC has been demonstrated to engage directly with the *NFE2L2* promoter, leading to the upregulation of Nrf2 expression and subsequently facilitating the malignant characteristics of cancer by altering nucleotide biosynthesis pathways 78. Notably, this regulatory mechanism may be of greater significance than Nrf2-mediated redox regulation. In recent years, numerous noncoding RNAs, such as microRNAs, circular RNAs, and long noncoding RNAs, have been extensively studied because of their involvement in modulating the Nrf2 pathway. Notable examples include miR-140-5p, miR-34a/b/c, circPIBF1, lncMALAT1, and lncMT1DP, which regulate Nrf2 expression or activity and thus play an important role in the development of various cancers and their responses to therapeutic interventions 79-83.

Nrf2 signaling is also influenced by epigenetic factors, including DNA methylation and chromatin modification. For example, in colon cancer, oxidative stress can induce DNA demethylation, leading to increased Nrf2 expression and resistance to 5-fluorouracil 84. In another independent study, the reduction of methylation at the *NFE2L2* promoter was found to enhance its expression, trigger apoptosis, and contribute to an anticancer effect 85. The function of Nrf2 is influenced by a combination of genetic and epigenetic factors, resulting in a complex impact on cancer. However, the precise mechanisms underlying them require further investigation. In addition, the impact of protein posttranslational modifications, including acetylation, sumoylation, and glycosylation, on Nrf2 signaling has received significant attention. In colon cancer, ARD1 has been demonstrated to acetylate Nrf2, thereby inhibiting its degradation via the proteasome pathway, further facilitating Nrf2 nuclear translocation, enhancing the transactivation of downstream target genes, and ultimately contributing to colon cancer cell proliferation and malignant phenotype 86. In the case of hepatocellular carcinoma (HCC), Nrf2 sumoylation was demonstrated to promote continuous HCC growth by enhancing the removal of intracellular reactive oxygen species (ROS) and regulating cellular metabolism, which plays a crucial role in the development of HCC and related metabolic stress 87. A study by Sanghvi *et al.* also revealed that the glycosylation of Nrf2 destabilizes the protein and impairs the ability of cancer cells to withstand ROS stress in a Keap1-dependent or Keap1-independent manner 88. Existing research has provided initial insights into the effects of various posttranslational modifications on the stability and activity of Nrf2. However, further investigation is warranted to elucidate the abundance, function, and regulatory mechanisms of each modification in different types of cancers.

### 3.3. Nrf2: tumor suppressor or oncogene?

In recent decades, a growing body of research has explored the diverse mechanisms involved in the regulation of Nrf2. These mechanisms include alternation in the canonical Nrf2-Keap1 complex, disruptions in noncanonical p62-dependent Nrf2-Keap1 interactions, and control of Nrf2 mRNA and protein expression through transcriptional and translational processes 89. It is worth noting that Nrf2 plays a dual role in the initiation and progression of cancer, with its effects varying in different conditions.

Traditionally, Nrf2 has been recognized as a significant regulator of detoxification and redox homeostasis, playing a crucial cytoprotective role. In response to stress, Nrf2 activates cytoprotective mechanisms by inducing the expression of multiple genes involved in antioxidant signaling, exogenous biotransformation, autophagy, and proteostasis 9, 90, 91. Research has demonstrated that when mice with mutated *NFE2L2* are exposed to carcinogens, organ damage is increased, particularly in the liver, kidneys, and lungs 92. Additionally, studies on mice lacking Nrf2 have shown inhibition of ARE-mediated genes, such as *GST*, *γ-GCS*, *NQO1*, *HO-1*, and *GCL*, which are involved in detoxification processes, leading to increased susceptibility to cancer 93. It is important to note that recent investigations have uncovered additional functions of Nrf2 in cancer that go beyond its role in redox regulation, including response to ER stress, growth factor signaling, and nutritional status 94-96. Therefore, Nrf2 is commonly acknowledged as a tumor suppressor, and the activation of Nrf2 signaling is employed in cancer chemoprevention to enhance cellular and systemic defenses against cancer 97. The activation of Nrf2-related pathways via Nrf2 inducers has been demonstrated to impede cancer progression and enhance the sensitivity of cancer cells to chemotherapeutic agents. Among the plant-derived Nrf2 inducers are compounds such as sulforaphane, curcumin, epigallocatechin gallate, lycopene, and resveratrol 80, 98-101. Chemically synthesized or derived substances, including indole and triterpenoid analogs, have also been identified as effective Nrf2 inducers 102, 103.

By contrast, in recent years, an increasing body of evidence has emerged suggesting that the activation of Nrf2 may not be advantageous for all types and stages of cancers. In fact, the activation of Nrf2 enhances the survival of not only normal cells but also cancer cells, supporting the notion that Nrf2 activation may play a role in the maintenance and advancement of cancer, as well as provide protection to tumor cells against oxidative damage that could potentially result in cell death 104-106. Numerous studies have demonstrated that cancer patients exhibit elevated levels of Nrf2 compared to individuals without cancer 104. The hyperactivation of Nrf2 in oncogenesis is frequently modulated by various factors. Apart from alterations in Nrf2/Keap1/p62/Cul3, the mutation and activation of oncogenes (e.g., KRAS^G12V/D^, B-RAF^V619E^, and MYC) or the inactivation of tumor suppressor genes (e.g., Trp53/p16) can also contribute to the hyperactivation of Nrf2 107, 108.

Subsequent studies have further confirmed the facilitation of Nrf2 in the initiation and progression of cancer. For instance, in lung cancer, the loss of Keap1 heterozygosity and mutations result in decreased Keap1 expression, leading to the upregulation of Nrf2 expression and the activation of its downstream genes 109, 110. Similarly, the Keap1^C23Y^ mutation identified in breast cancer reduces its ability to inhibit Nrf2 expression 111. In the unstable oxidative microenvironment of hypoxia/reoxygenation, cancer cells also exhibit reduced Keap1 expression, which promotes nuclear translocation and increased expression of Nrf2, which, in turn, contributes to the removal of ROS and the progression of cancer 112. In summary, the diminished expression and functional loss of Keap1 may result in the sustained activation of Nrf2, thereby providing growth support for cancer cells.

Chemotherapy resistance poses a significant obstacle to the clinical management of cancer. Recent research has indicated that Nrf2 may play a role in chemotherapy resistance. Specifically, there is a positive association between Nrf2 levels and the resistance of cancer cells to chemotherapy drugs, such as cisplatin, doxorubicin, and etoposide 69, 113. Cancer cell resistance to drugs is heightened when Nrf2 expression is increased. For instance, Xu *et al.* established that in HNSCC, there is an obstruction to Nrf2 degradation mediated by the ubiquitin-proteasome pathway, leading to Nrf2 accumulation, which may contribute to the development of acquired cisplatin resistance in HNSCC 113. Another independent study by Lee *et al.* found that Nrf2 is a key regulator of cell survival in ovarian cancer cells under conditions of GSH depletion 105. Inhibiting Nrf2 using the GSH inhibitor L-buthionine-(S, R)-sulfoximine as a chemical sensitizer renders ovarian cancer cells more susceptible to doxorubicin. The Nrf2 pathway is also activated in doxorubicin and etoposide-resistant osteosarcoma cells 69. DDRGK1 knockdown results in increased Nrf2 degradation and decreased Nrf2 stability, leading to an increase in ROS levels, which subsequently facilitates the apoptosis of cancer cells and heightens their sensitivity to chemotherapeutic agents, such as doxorubicin and etoposide. Combining DDRGK1 knockout with chemotherapy may yield enhanced therapeutic outcomes in the treatment of osteosarcoma. In conclusion, targeting Nrf2 and related pathways may offer novel strategies and avenues for reversing chemotherapy resistance in cancer.

## 4. Crosstalk between autophagy and Nrf2 signaling

**Figure 3:**
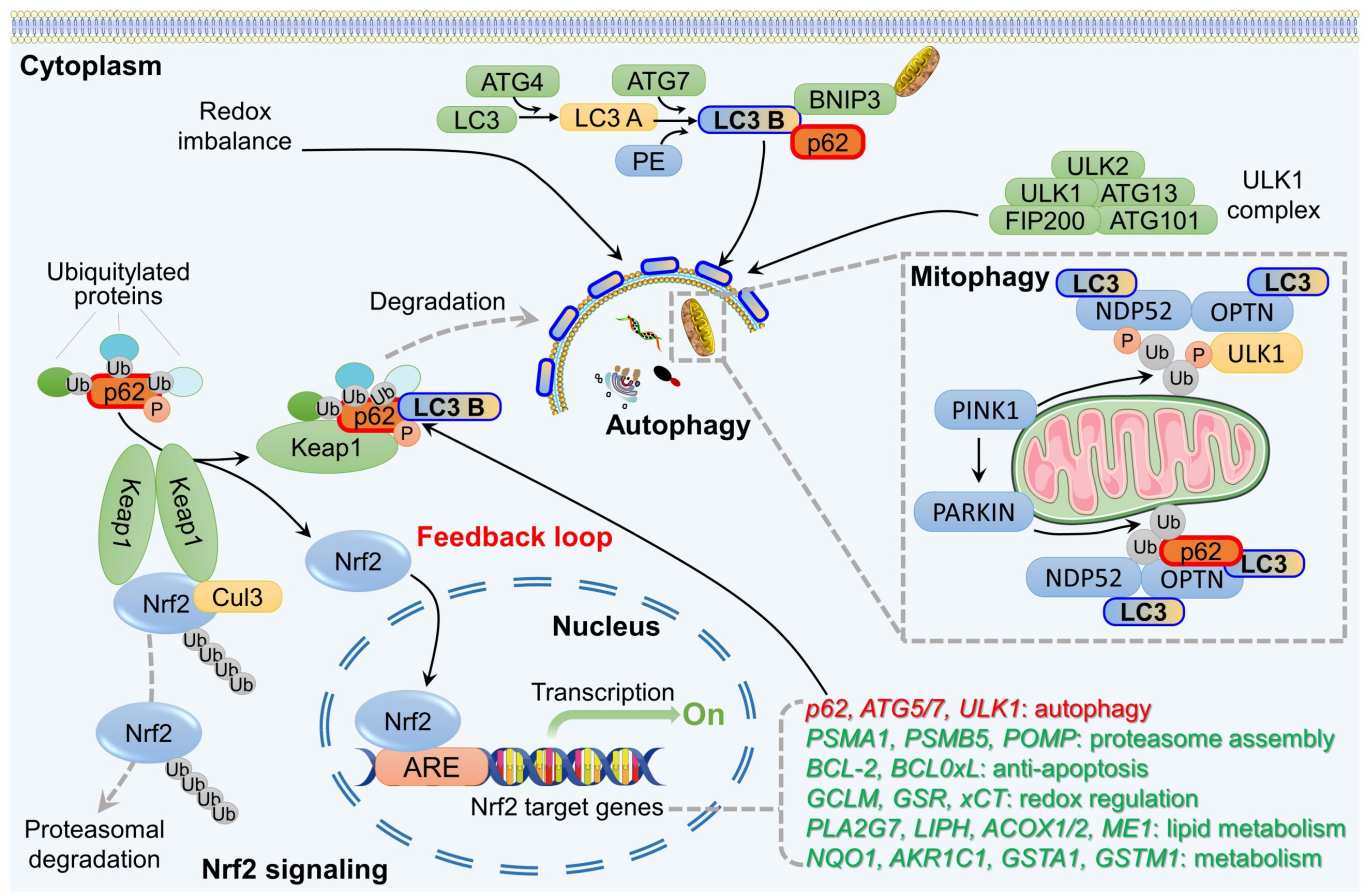
Crosstalk between the autophagy and Nrf2 pathway. P62 is a receptor protein involved in autophagy that binds to ubiquitin and serves as a key link between the Nrf2 pathway and autophagy. Through post-translational phosphorylation, p62 exhibits an increased affinity for Keap1, leading to the dissociation of Keap1 from Nrf2. This process results in the formation of p62-Keap1 heterodimers that recruit LC3, facilitating selective autophagic degradation of Keap1. As a result, Nrf2 accumulates in the cytoplasm, translocates to the nucleus, and activates transcription of various downstream genes. In addition, Nrf2 promotes p62 expression, establishing a positive feedback loop involving p62-Keap1-Nrf2. In particular, PINK1 localizes to the outer membrane of damaged mitochondria, recruiting PARKIN from the cytoplasm to damaged mitochondria, where PARKIN is phosphorylated. Phosphorylated PARKIN then ubiquitinates p62, facilitating its binding to LC3 B and promoting the recruitment of damaged mitochondria for degradation through mitophagy. p62, NDP52, and OPTN act as mitophagy-associated adaptors.

A comprehensive understanding of the interplay between the Nrf2 signaling pathway and autophagy has been elucidated by several research groups 12, 35, 98, 114, 115. These investigations simultaneously unraveled the mechanism underlying the connection between Nrf2 and various autophagy receptors, the functional implications of autophagy dysregulation, and the regulation of the persistent activation of the Nrf2 signal. Among these, p62, which functions as a scaffold protein and stress-inducing protein and plays a role in multiple biological processes, including autophagy, apoptosis, inflammation, cell survival, cell death, signal transduction, and tumorigenesis, has been extensively investigated 13, 114, 116. Simply put, Nrf2 regulates the transcription of ATGs and, at the same time, stimulates autophagy directly through nontranscriptional pathways. The autophagy adaptor protein p62, in turn, is involved in the noncanonical activation of Nrf2. These findings suggest the existence of a positive feedback loop between Nrf2 signaling and autophagy (Figure 3).

### 4.1. The p62-Keap1-Nrf2 feedback loop

p62 is recognized as the first selective autophagy receptor and serves a significant regulatory function within the autophagy pathway. Through its interaction with LC3, p62 attaches to the autophagosome, facilitating the transport of ubiquitinated cargo to the lysosome for degradation 16. It has been reported that p62 is implicated in the regulation of the Nrf2 pathway, leading to Nrf2 translocation to the nucleus and subsequent activation of the transcription of antioxidant enzyme genes 13. This process involves several main steps. First, during the oxidative stress response, the accumulation of p62 protein resulting from autophagy dysfunction leads to the interaction between p62 and Keap1, which sequesters Keap1 within p62 aggregates 117. Research has demonstrated that when cells are exposed to ROS, phosphorylation of the KIR domain of p62 significantly enhances binding affinity with the DGR domain of Keap1 118. Subsequently, p62 binds to LC3 through the LIR domain, forming the LC3-p62-Keap1 complex, which transports Keap1 to autophagosomes for degradation 119. This binding reduces the formation of the Keap1-Cul3-E3 complex and reduces its degradation through the ubiquitin-proteasome pathway, resulting in the activation of Nrf2 signaling.

Interestingly, Nrf2 may participate in the autophagy pathway, either directly or indirectly. On the one hand, Nrf2 interacts with ARE located in the promoter region of the *p62* gene, thereby facilitating p62 transcriptional activation 120. That is, *p62* is recognized as a target gene for Nrf2. On the other hand, overexpression of p62 may enhance Nrf2 activity by promoting Keap1 degradation and decreasing Nrf2 ubiquitination. This establishes an Nrf2-Keap1-p62 positive feedback loop that sustains Nrf2 activation 121. This evidence indicates that p62, acting as a regulatory interactor of the Nrf2-Keap1 complex, is present within the cellular milieu alongside other interactors and regulators that participate in various mechanisms to modulate the unbound and active state of this transcription factor. Ultimately, these processes facilitate its degradation in the cytoplasm and nucleus, thereby regulating or potentially stopping Nrf2-dependent stress responses.

The interaction between p62 and Nrf2 in this feedback loop is influenced by various factors and holds significant importance in cellular antioxidant response and the development and treatment of cancer. For instance, Shi *et al.* demonstrated that inhibition of autophagy mitigates p62-mediated Keap1 sequestration, subsequently reducing the Nrf2-mediated transcriptional activation of antioxidant genes, which ultimately facilitates the initiation and progression of prostate cancer 13. Similarly, autophagy activation in gastric cancer significantly enhances the interaction between p62 and Keap1 while simultaneously inhibiting the binding of Nrf2 to Keap1, which reduces the ubiquitination and degradation of Nrf2; this results in a sustained activation of the Nrf2 signaling pathway and inhibition of malignant progression 122. In addition, investigations into the anticancer mechanisms of apatinib, a targeted angiogenesis inhibitor, have revealed that it can induce ROS production in lung cancer, suppress Nrf2 and p62 expression, and promote autophagy and apoptotic cell death in non-small cell lung cancer (NSCLC) 123. This mechanism is considered one of the critical pathways through which apatinib exerts its antitumor effects, both *in vitro* and *in vivo*. In summary, the Nrf2 signaling pathway and autophagy exhibit mutual regulatory interaction through the p62-Keap1-Nrf2 positive feedback loop, and they perform distinct pathological and pharmacological functions across various cancer contexts.

### 4.2. Effect of the Nrf2-p62 loop on mitophagy

The association between Nrf2 and mitophagy was initially identified because of Nrf2's function as a transcriptional regulator. Murata *et al.* discovered that Nrf2 can bind to four AREs in the promoter region of the *PINK1* gene, thereby influencing its transcription and subsequent expression 124. Specifically, the overexpression of Nrf2 leads to an increase in PINK1 expression, whereas the suppression of Nrf2 has the opposite effect. These pieces of evidence support the idea that Nrf2 induces PINK1 to participate in mitophagy. Nrf2 has also been found to participate in the modulation of mitophagy independently of the PINK1/PARKIN pathway. Gumeni *et al.* discovered that in a fruit fly model lacking PINK1/PARKIN, the activation of Nrf2 can stimulate mitophagy and effectively mitigate and reverse neurodegenerative alterations resulting from PINK1/PARKIN suppression 125. This suggests that alternative mechanisms could potentially exist through which Nrf2 regulates mitophagy, necessitating additional investigation.

The involvement of p62 in mitophagy is also linked to PINK1/PARKIN. PARKIN possesses E3 ubiquitin ligase activity, enabling it to ubiquitinate many proteins located on the outer membrane of mitochondria and subsequently triggering mitophagy 126. PARKIN facilitates K63 polyubiquitination of mitochondrial substrates and recruits the ubiquitin and LC3 binding protein p62 to mitochondria 127. p62 can interact directly with ubiquitinated molecules on autophagosomes, and p62 knockout completely blocks the clearance of damaged mitochondria. Therefore, activation of the PINK1/PARKIN/p62 axis plays a critical role in mitophagy, which is essential for maintaining mitochondrial quality control and homeostasis. However, a recent challenge to this conventional model has emerged, as PINK1 has been found to recruit two additional adaptors, OPTN and NDP52, instead of p62 128. This recruitment leads to the activation of the key autophagy serine/threonine kinase ULK1 and the induction of mitophagy. Notably, this process occurs independently of PARKIN. Comprehending the role of Nrf2 in mitophagy and autophagy is critical to gain insight into the mechanisms by which Nrf2/p62 regulates mitochondrial quality control and maintains mitochondrial homeostasis.

## 5. Implication of Nrf2-autophagy crosstalk in cancer progression and therapeutics

A growing body of research has indicated that p62 and several ATGs, including ATG5 and ATG7, are potential targets of Nrf2, indicating a close association between Nrf2 and autophagy 35, 129, 130. Investigating the connection between Nrf2-related autophagy and cancer development warrants further investigation and may provide a scientific basis for the formulation of cancer treatment approaches that target Nrf2.

### 5.1 Effects of Nrf2-autophagy crosstalk on cancer progression and therapy response

As previously noted, the *dark side* of Nrf2 in cancer has gained significant interest since 2006 131. In adverse conditions, elevated levels of Nrf2 and activated autophagy within cancer cells foster a favorable cellular environment that facilitates proliferation and survival, thereby exacerbating cancer progression, recurrence, and metastasis. In addition, Nrf2-autophagy crosstalk contributes to the development of resistance to chemotherapy to some extent.

HCC is a widespread form of malignancy worldwide and ranks as one of the most common causes of cancer-related mortality. Recent research has revealed that the crosstalk between Nrf2 signaling and autophagy can affect the advancement of HCC. Komatsu *et al.* presented initial evidence of the involvement of the Nrf2-p62 axis in the development of HCC 132, 133. Elevated p62 levels in livers with impaired autophagy sustain the progression of precancerous lesions and HCC. In particular, excessive p62 competes with the Nrf2 binding site on Keap1, leading to the stabilization of Nrf2 and the subsequent activation of Nrf2 target genes. In addition to p62 overexpression, p62 phosphorylation has been observed in HCC, both of which contribute to the activation of Nrf2 134, 135.

Nrf2-autophagy crosstalk has been extensively investigated in various types of cancers. For instance, in prostate cancer, speckle-type POZ protein has been observed to bind to and trigger the nondegradable ubiquitination of p62, resulting in the inhibition of p62-dependent autophagy 13. Consequently, this process leads to Keap1 isolation, ultimately reducing the transcriptional activation of Nrf2-mediated antioxidant genes, which in turn facilitates prostate cancer progression. Similarly, BDH2 facilitates the ubiquitination of Nrf2 by enhancing the interaction between Keap1 and Nrf2 in gastric cancer, leading to increased ROS accumulation, activation of cellular autophagy, and inhibition of gastric cancer progression 122. Nrf2 has also been identified as an independent prognostic factor influencing overall survival in patients with NSCLC 136. Notably, Nrf2 stimulates the formation of autophagosomes, thereby promoting the progression of NSCLC. Conversely, regulators inhibit the Nrf2-autophagy axis to suppress cancer progression. In osteosarcoma, TRIM22 has been observed to promote Keap1-independent degradation of Nrf2, thereby activating autophagy signaling pathways and ultimately impeding osteosarcoma progression 60. This suggests that the TRIM22/Nrf2/autophagy signaling axis may represent a promising therapeutic target for osteosarcoma treatment. In general, these studies offer new perspectives on the comprehension of the Nrf2-autophagy signaling axis in carcinogenesis and development, and they have the potential to advance the creation of novel cancer treatment approaches.

### 5.2 Agents targeting the Nrf2-autophagy signaling axis in cancer therapy

The dynamic role of Nrf2-autophagy crosstalk in cancer raises the question of whether its regulation is beneficial to cancer patients. A growing body of research has demonstrated that both natural and synthetic ligands can modulate the Nrf2-autophagy signaling axis through direct or indirect means, thus affecting the progression and resistance to treatment of various types of cancers (Table 2). Recent investigations have also utilized a combination of antitumor drugs and regulators of the Nrf2-autophagy signaling axis to uncover the role of the Nrf2-autophagy signaling axis in the onset and progression of cancer.

Medicinal plants contain a wealth of potential treatments for various diseases. Researchers have discovered that a range of natural compounds and their derivatives can affect the Nrf2-autophagy signaling axis, leading to anticancer properties. For example, sulforaphane, an isothiocyanate compound obtained from cruciferous plants, has been shown to affect cancer progression by modulating various cellular processes, such as proliferation, metastasis, apoptosis, and angiogenesis 137. Recent research has indicated a strong correlation between the pharmacological effects of sulforaphane and the Nrf2-autophagy signaling axis. In cervical cancer, sulforaphane has been found to induce p62 expression and p62 body formation through the transcriptional coactivator SPBP, thereby influencing the activation of cytoprotective autophagy signaling associated with Nrf2 138. In lung cancer, the triterpenoid glycoside oleifolioside B and sesquiterpene lactone compound isodeoxyelephantopin (ESI) have been found to stimulate cytoprotective autophagy 139, 140. In particular, ESI prompts nuclear translocation of Nrf2 and triggers p62 transcription, which subsequently competes with Keap1 for binding, releasing Nrf2 and establishing a positive feedback loop for p62 activation. Regarding its anti-lung cancer properties, ESI demonstrates the selective inhibition of both the viability and clonogenic potential of lung cancer cell lines while exhibiting minimal toxicity to lung epithelial cells.

Targeting the Nrf2-p62-Keap1 regulatory axis combining ESI presents a potentially promising therapeutic approach for the fight against lung cancer. Moreover, research has demonstrated that lycopene can promote the activation of intracellular antioxidant enzymes and the nuclear translocation of Nrf2 100. Lycopene has also been found to increase p62 expression, leading to Keap1 degradation and the subsequent release of Nrf2. Subsequent research has demonstrated that lycopene exhibits a protective effect against the development of cutaneous papilloma in both cellular and animal models. These findings elucidate the mechanistic connection between lycopene-induced Nrf2-autophagy signaling and provide potential preclinical support for chemoprevention of skin cancer. The above research has provided promising prospects for identifying precursors targeting the Nrf2-autophagy signaling axis from natural products, which may serve as a foundation for further investigation and theoretical underpinning in the advancement of innovative cancer treatment approaches.

Chemically synthesized drugs play a crucial role in the treatment of cancer because of their rapid therapeutic action and evident efficacy. Certain small-molecule compounds and drugs have demonstrated the ability to affect the Nrf2-autophagy axis, resulting in promising anticancer properties. For instance, jaspine B derivative C-2 exhibits selective inhibition of gastric cancer cell proliferation compared to normal epithelial gastric cells 121. Interestingly, C-2 induces autophagy and enhances p62 expression in gastric cancer cells by activating the JNK/ERK/Beclin 1 pathway. Consequently, p62 competitively binds to Keap1, leading to the release of Nrf2 and its translocation to the nucleus, thereby promoting the expression of downstream Nrf2 target genes and enhancing gastric cancer cell survival. These findings indicate that gastric cancer cells may activate protective autophagy via the p62/Keap1/Nrf2 pathway in the early stages to counteract C-2-induced cell death. Moreover, in NSCLC, apatinib has been observed to induce the generation of ROS, suppress the expression of Nrf2 and p62, trigger autophagy and apoptosis, and inhibit tumor proliferation, both *in vitro* and *in vivo* 141. In a separate independent investigation, Yu *et al.* elucidated the cytotoxic properties of C-2 in the context of bladder cancer 142. Mechanistically, C-2 activates the JNK pathway, which subsequently induces autophagy and increases p62 expression. C-2 also activates the Nrf2 signaling pathway, facilitates the transition from autophagy to apoptosis, and amplifies the apoptotic effect on cancer cells. Notably, the coadministration of C-2 with the JNK inhibitor SP600125 further enhances the tumor-suppressive effects of C-2, potentially because of the promotion of apoptosis and the attenuation of autophagy. These results provide a basis for further research and theoretical justification for the development of pharmacological agents that target the Nrf2-autophagy signaling axis.

The combination of small-molecule compounds or medications presents novel prospects for the prevention and management of cancer. Particularly, focusing on the Nrf2-autophagy axis has significant implications for overcoming resistance to cancer treatment. Colon cancer is a prevalent and deadly malignant tumor. mTOR inhibitors, including rapamycin, have been employed in the treatment of colon cancer, but the development of drug resistance frequently results in cancer evasion 143. Li *et al.* investigated the potential of S-allylmercaptocysteine (SAMC) as an adjuvant to rapamycin in the treatment of colon cancer, with a particular focus on the interplay between Nrf2 and autophagy in the context of combination therapy 144. The researchers found that the combined application of rapamycin and SAMC stimulated the transcription of Nrf2 and its downstream *NQO1* gene while suppressing p62 expression. In terms of effectiveness, the combined treatment of SAMC and rapamycin has the potential to enhance the anticancer efficacy. These results provide novel insights into colon cancer treatment and suggest that the Nrf2-autophagy pathway could represent a promising new therapeutic target for colorectal cancer. The combination of sulforaphane with other pharmacological agents has also demonstrated significant anticancer efficacy across various malignancies, including prostate cancer, breast cancer, and esophageal cancer, among others. In particular, the combination of sulforaphane and vitamin D has been shown to diminish cell viability in prostate cancer by inducing oxidative stress, DNA damage, and autophagy, which is linked to the upregulation of several key proteins, including BAX, CASP8, CASP3, JNK, and Nrf2 145. Furthermore, in esophageal squamous cell carcinoma, sulforaphane promotes autophagy through the activation of the Nrf2 pathway, which subsequently inhibits the proliferation and clonogenic potential of cancer cells 115. The use of the autophagy inhibitor chloroquine (CQ) has been found to inhibit the Nrf2 pathway while activating the caspase pathway, thereby enhancing the antitumor efficacy of sulforaphane in both *in vitro* and *in vivo* models. These findings provide a preclinical theoretical framework for the potential application of sulforaphane in future cancer treatment strategies.

The Nrf2-autophagy signaling pathway presents novel pharmacodynamic targets for certain anticancer drugs that are currently utilized in clinical practice. For instance, apatinib is a prominent antiangiogenic drug that demonstrates significant inhibitory effects on various solid tumors, including NSCLC 146. Beyond its traditional mechanism of targeting the VEGFR2/STAT3 signaling pathway, apatinib has been found to enhance ROS generation, suppress Nrf2 and p62 expression, and subsequently induce autophagy and apoptotic cell death in NSCLC 141. Similarly, in the context of breast cancer, apatinib has been observed to downregulate the Nrf2/HO-1 signaling pathway and suppress glutathione levels; this leads to ROS generation, which facilitates ROS-dependent autophagy and apoptosis, and ultimately results in the inhibition of both the proliferation and migration of breast cancer cells 147. These findings enhance the pharmacodynamic investigation of apatinib and are expected to contribute to the broader clinical utilization of the drug. Moreover, temozolomide is a chemotherapeutic agent employed in the treatment of malignant glioma and malignant melanoma 148, 149. Recent research indicates that downregulation of Nrf2 may exacerbate autophagy and the proliferation suppression of temozolomide-induced glioma cells 150. Future investigation into the synergistic effects of Nrf2 inhibitors in combination with temozolomide may enhance therapeutic outcomes for glioma, requiring further pharmacological exploration. Furthermore, procaine is a widely utilized local anesthetic in clinical settings 151. Recent studies have demonstrated that in oral squamous cell carcinoma (OSCC), procaine enhances the expression of the differentiation gene PAX9 and activates the Nrf2 signaling pathway, which subsequently leads to the inhibition of OSCC cell proliferation, differentiation, and stemness via an autophagy-dependent mechanism 152. Elucidating the pharmacological effects and molecular mechanisms of procaine may offer a novel therapeutic approach for the treatment of OSCC. In conclusion, these findings indicate that the Nrf2-autophagy signaling axis holds promise as a target for anticancer therapy. Elucidating the mechanism of action of the Nrf2-autophagy signaling axis may offer a novel avenue for drug development.

### 5.3. Nrf2-p62 and other autophagy-related proteins in cancer monitoring

For a long time, the prompt detection of cancer and the evaluation of treatment effectiveness have been key components of clinical practice. Extensive efforts have been made to identify markers that can help predict cancer risk and establish preventive measures. These markers play a pivotal role in facilitating timely diagnosis and prognostic assessment, thus influencing the selection of clinical treatment options and secondary prevention strategies. Because of the involvement of the Nrf2-autophagy signaling axis in tumorigenesis and cancer resistance, significant efforts have been made to identify Nrf2-p62 and other autophagy-related markers for predicting cancer risk, establishing preventive measures, and facilitating timely diagnosis and prognosis assessment of cancer (Table 3).

Research has indicated that elevated levels of Nrf2 can facilitate cancer cell survival, enhance resistance to chemicals, and function as a prognostic indicator for various malignancies, including lung cancer, cervical cancer, pancreatic cancer, and breast cancer 153-156. Additionally, p62 aggregation has been linked to an unfavorable prognosis in triple-negative breast cancer, lung adenocarcinoma, and glioma 157-159. In this context, Nrf2, p62, and other autophagy-related proteins have been suggested and further investigated for their potential utility in clinical protocols for assessing cancer risk, establishing prognostic scores, and determining intervention criteria for cancer patients. In basic terms, prolonged elevation of Nrf2 expression and heightened activity increases the likelihood of developing precancerous lesions and cancers during the carcinogenesis stage. Conversely, in later stages of cancer, it is associated with a negative prognosis. Sustained activation of the Nrf2 pathway through increased autophagy signaling, particularly p62-mediated activation, may provide valuable clinical insights into the severity of precancerous lesions and associated cancer risk. Furthermore, the cancer-specific reprogramming of metabolic and stress response pathways prompted by p62-mediated activation of the Nrf2 pathway warrants further exploration as a potential prognostic and therapeutic indicator for cancer.

In addition, other proteins associated with autophagy may contribute to advancing and supporting the clinical utilization of the Nrf2-p62 pathway in cancer surveillance. For instance, LC3 can be valuable in distinguishing cancer progression and conducting prognostic assessment. LC3 A exhibits high expression in HCC tissues 160. Significantly, LC3 A has a *stone-like* appearance in HCC, and its expression level correlates with elevated serum alpha-fetoprotein levels, a lower degree of tumor differentiation, and vascular invasion. In addition, elevated levels of stone-like LC3 A are strongly associated with unfavorable outcomes for HCC patients, thus serving as an independent prognostic factor. Conversely, LC3 B expression is closely related to a large tumor size, advanced tumor stages, and worse relapse-free and overall survival, making it a potential molecular marker for guiding the selection of cancer staging and surgical treatment options 161.

In mammals, alongside LC3, a group of autophagy-associated proteins that are homologous to yeast ATG8 belongs to the gamma-aminobutyric acid type A receptor-associated protein (GABARAP) family (e.g., GABARAP, GABARAPL1, and GABARAPL2). These proteins are reported to engage with molecules involved in vesicle transport, autophagy, and programmed cell death, serving a crucial function in regulating autophagy initiation and autophagosome closure 218. Studies have shown a decrease in the expression of GABARAP and GABARAPL1 in different types of tumor tissues. For instance, in the context of breast cancer, decreased GABARAP levels have been observed to trigger EMT and facilitate tumor progression 214. In addition, there exists an inverse correlation between GABARAP expression and advanced clinicopathological characteristics (e.g., tumor size and tumor node metastasis (TNM) stage) in clinical samples. Patients with reduced GABARAP levels tend to have an unfavorable prognosis. In summary, GABARAP has the potential to hinder the malignant progression of breast cancer and may serve as a promising target for diagnosis and therapeutic intervention. In a separate investigation, Gil *et al.* observed a significant decrease in GABARAP expression in colorectal cancer tissue 215. GABARAP functions as a tumor suppressor in the development of colorectal cancer. A reduced GABARAP expression is associated with inferior differentiation of colorectal cancer and shorter overall survival, indicating its potential as a therapeutic target for colorectal cancer treatment. Moreover, Brigger *et al.* conducted clinical research demonstrating a notable decrease in the expression of GABARAP family members GABARAPL1 and GABARAPL2/GATE-16 in individuals diagnosed with primary acute myeloid leukemia 216. Specifically, diminished levels of GABARAPL2 were linked to immature myeloid leukemia phenotypes. A reduced expression of these GABARAP family proteins is essential for early neutrophil differentiation in leukemia. However, the levels of GABARAP expression in various tumors are inconsistent. For example, GABARAP and GABR2 are notably increased in thyroid adenoma and early thyroid cancer tissues, and they contribute to tumor growth and spread 217. This illustrates the intricate functional role of GABARAP in tumor development and highlights the complexity of the mechanisms underlying cancer initiation and progression. Further investigations are warranted to support these findings in the future.

UVRAG, a crucial regulator of macrophage/autophagy in mammals, interacts with Beclin 1, PIK3C3, and RUBCN. Shi *et al.* found that UVRAG has the potential to increase tumor migration and drug resistance, resulting in a negative prognosis for individuals with colorectal cancer 162. These findings provide a basis for the development of treatment strategies for colorectal cancer patients. Moreover, research has indicated that UVRAG phosphorylation plays a negative role in autophagosome maturation. Specifically, phosphorylation levels at UVRAG S522 have been associated with a less favorable prognosis in patients with HCC, and further research is warranted to determine its clinical application value 163.

Moreover, ULK1 is a serine/threonine kinase that is recognized for its role in autophagy initiation. Wu *et al.* highlighted the significance of ULK1 as a crucial prognostic factor in patients with HCC 164. The simultaneous incorporation of LC3 B in assessments can also enhance the accuracy of prognosis and survival predictions for these patients. In the context of breast cancer, ULK1 is strongly associated with disease progression and adverse prognostic outcomes, suggesting its potential as a novel therapeutic target and prognostic marker for breast cancer 165. Furthermore, Yun *et al.* investigated ULK1 expression patterns and prognostic implications in nasopharyngeal carcinoma 166. Their findings revealed a strong correlation between elevated ULK1 levels and aggressive clinical features, as well as poor survival rates in individuals with nasopharyngeal carcinoma.

Furthermore, Beclin 1 plays a crucial role in the assembly of a significant protein complex within the autophagy pathway, contributing to various stages including autophagosome initiation, elongation, and maturation 1. A reduced Beclin 1 expression is associated with impaired autophagy in cancer, making it a significant biomarker associated with carcinogenesis and progression. Its reduced expression is believed to impact the prognosis and clinical outcomes of cancer patients. Du *et al.* found that NSCLC patients with reduced levels of Beclin 1 were diagnosed at a later stage, had a higher incidence of lymph node metastases, and exhibited lower tumor differentiation 167. Consequently, Beclin 1 may offer more precise prognostic indicators for NSCLC patients, potentially benefiting a larger number of individuals with this condition. Similarly, a decreased expression of Beclin 1 in gastric cancer correlates with lymph node metastasis, TNM staging, dedifferentiation, and adverse prognosis 168. In an independent study by Dong, it was observed that a decreased Beclin 1 expression was markedly linked to lymph node metastasis in intrahepatic cholangiocarcinoma, as well as to reduced overall survival and disease-free survival 169. The expression of Beclin 1 is correlated with the advancement and spread of intrahepatic cholangiocarcinoma, suggesting its potential utility as a novel prognostic marker for patients. Together, Beclin 1 holds promise as a potential indicator for assessing the onset, invasion, metastasis, and prognosis of cancer, and may serve as a target for gene therapy in cancer.

Advances in technology and research have led to the discovery of an increasing number of autophagy-related proteins that significantly affect cancer prognosis and clinical medication monitoring. Utilizing autophagy-related proteins as biomarkers for cancer surveillance provides a thorough understanding of the interplay between autophagy and cancer, offering novel perspectives on cancer treatment strategies that focus on autophagy modulation.

### 5.4. Ongoing clinical trials of Nrf2 and autophagy signaling

Currently, the Clinical Trials Database (https://www.clinicaltrials.gov) contains approximately 130 records targeting Nrf2 and autophagy in cancer. The trials focused on advancing innovative anticancer drugs that specifically target Nrf2 and autophagy and on exploring the potential of combining established drugs. Given its significance as a regulatory element in cancer and a target for therapeutic measures, Nrf2 and autophagy signaling have attracted considerable interest in both preclinical and clinical investigations.

Clinical trials have been conducted to assess the safety, tolerability, pharmacological properties, and antitumor effects of Nrf2 inhibitors, including pyrimethamin (NCT05678348) and sulforaphane (NCT03182959), as well as the GLS1 inhibitor telaglenastat (NCT03872427, NCT04265534), TORC 1/2 inhibitor sapanisertib (NCT02417701), Keap1 activator VD-130037 (NCT05954312), and other compounds in Nrf2-mutated cancer types, such as HNSCC, NSCLC, and acute myeloid leukemia (Table 4). For instance, sapanisertib, a TORC1/2 inhibitor, has shown enhanced therapeutic efficacy and extended progression-free survival in NSCLC models and phase II clinical trial populations characterized by Nrf2 activation 170. In combination with glutaminase inhibitors, it may also help reduce metabolic resistance to treatment in patients. These findings indicate that modulation of the Nrf2 pathway, in conjunction with metabolic targeting, could yield promising therapeutic strategies for NSCLC patients lacking genotype-directed therapies. A limited number of clinical trials have focused on Nrf2 as a potential target for anticancer treatment. As interest in Nrf2-targeted cancer therapies grows, there is a need for the continued development of improved Nrf2 modulators to enhance clinical efficacy.

Increased autophagy is believed to be the mechanism by which cancer cells survive and develop resistance to chemotherapy. It has been widely demonstrated that inhibiting autophagy can sensitize cancer cells to anticancer treatments. Small-molecule inhibitors of autophagy can be used independently or in combination with anticancer drugs. Currently, CQ/hydroxychloroquine (HCQ) is the only FDA-approved autophagy inhibitor for clinical trials 18. CQ, an anti-malarial drug, inhibits autophagosome-lysosome fusion by affecting lysosomal acidification. Numerous studies have shown that CQ can enhance the sensitivity of cancer cells to chemotherapy drugs and even reverse resistance to chemotherapy 15, 171, 172. Presently, the combination of CQ/HCQ with various chemotherapy drugs is undergoing clinical trials (Table 4). The development of drug resistance in cancer presents a significant challenge in cancer treatment. The potential use of autophagy inhibitors in combination with chemotherapy drugs holds promise for addressing this problem, particularly in recurrent and refractory cancer types.

## 6. Conclusion and perspective

The Nrf2-Keap1 pathway and autophagy are critical mechanisms that contribute to cellular resistance to various stimuli, primarily by increasing the expression of a range of antioxidant and cellular defense genes 109. Normally, periodic activation of Nrf2 via the conventional pathway provides cellular security and operational efficiency. By contrast, continuous activation of Nrf2 promotes tumor growth and contributes to chemotherapy resistance because of genetic mutations in Nrf2-controlled genes 9, 69, 91, 173. Autophagy is a cellular stress response essential for cellular homeostasis and organelle health 39. Recent research has demonstrated a close relationship between Nrf2 and the autophagy adaptor protein p62, highlighting a mechanistic connection that supports the sustained activation of Nrf2 signaling 129. In the context of oxidative stress, p62 facilitates Keap1 aggregation, leading to the stabilization of Nrf2 and the upregulation of ARE regulatory genes, including p62 itself. This interaction results in the establishment of an Nrf2-Keap1-p62 regulatory loop that plays a significant role in the regulation of cellular processes in cancer cells.

Targeting the Nrf2-autophagy signaling axis through either biological or pharmacological interventions may offer potential strategies for chemoprevention and the treatment of cancer. This paper provides a comprehensive overview of and recent advances in the functions and mechanisms of autophagy and the Nrf2 pathway, as well as their crosstalk in cancer progression. It also highlights the potential utility of targeting the Nrf2-autophagy signaling axis for cancer monitoring and therapeutic interventions. While further investigation is needed to fully understand this process, these findings suggest a promising avenue for the development of cancer therapies.

Despite advancements in understanding Nrf2, numerous questions remain to be addressed. For example, Nrf2 functions as a multifaceted transcription factor that regulates the expression of various genes implicated in cellular redox homeostasis, cell proliferation, apoptosis, inflammatory responses, and DNA repair, among others 104. However, it is important to note that not all genes regulated by Nrf2 influence autophagy uniformly. The precise molecular mechanisms through which specific stimuli activate Nrf2 signaling to modulate autophagy have not yet been fully elucidated, and the protein interaction networks that mediate responses to diverse signals are still poorly understood. Numerous studies have also highlighted the involvement of Nrf2 in autophagy, and there remains a lack of clarity in distinguishing between the transcriptional and nontranscriptional roles of the Nrf2 signaling pathway in this context. Consequently, subsequent research endeavors may need to focus on elucidating these uncertainties to enhance the comprehension of Nrf2. Such insights could prove instrumental in establishing the targetability of Nrf2 in cancer and formulating intervention strategies that specifically target Nrf2. Furthermore, the limited number of ligands that specifically target Nrf2 is currently undergoing initial phases of clinical trials, and the overall quantity of pharmacological agents aimed at the Nrf2 signaling pathway remains constrained. Consequently, there is significant potential for further pharmacological investigation in this area.

Existing data suggest that the inhibition of autophagy through the use of ligands or drugs demonstrates promising clinical efficacy in cancer therapy. Recent studies have also highlighted the beneficial synergistic effects of ligands that target the autophagy pathway when used in conjunction with chemotherapeutic drugs. For instance, in preclinical models, autophagy inhibitors have proven to be effective adjuncts to various cytotoxic drugs and targeted therapies, which enhance the sensitivity of cancer cells to chemotherapy and mitigate resistance mechanisms 15, 171, 172. Furthermore, clinical trials are increasingly exploring a range of combination therapies that incorporate both anticancer agents and autophagy inhibitors. Nonetheless, a comprehensive analysis and investigation into the selection of suitable autophagy inhibitors and chemotherapy agents are needed, as well as an understanding of their functions across various cancer treatment modalities, which is essential for identifying optimal combination therapies and their respective indications.

## Figures and Tables

**Table 1:** Roles and mechanisms of autophagy in cancer progressions.

Effect of autophagy on cancer progressions	Cancer progressions	Mechanisms
Promote	Cancer development	Metabolic adaptation: The proliferation and survival of tumors leads to an elevated requirement for cellular energy and anabolic precursors. Autophagy recycles intracellular components to provide substrates for metabolic and biosynthetic pathways, helping to mitigate the constraints imposed by the scarcity of external nutrients, thereby supporting and enhancing tumor progression^28^. Reduction of oxidative stress: Cancer cells exhibit increased levels of ROS as a result of mitochondrial dysfunction^174^. The accumulation of excessive ROS can induce oxidative stress, which subsequently results in damage to cellular components and ultimately leads to cell death. In response to the production of ROS and the presence of oxidative stress, autophagy is activated, indicating its potential role in exerting antioxidant effects and facilitating the survival of cancer cells^175^. Provide nourishment in the TME: Host cells supply essential metabolites, particularly amino acids, that are crucial for the growth and survival of cancer cells via the process of autophagy^44^; Autophagy plays a significant role in the regulation of immune evasion, antigen presentation, and the infiltration of immune cells, all of which have implications for tumor immunity^176^.
	Cancer metastasis	Autophagy facilitates the adaptation of pre-metastatic cells to heightened metabolic demands, oxidative stress, and challenging environmental conditions^177^. The detachment of ECM triggers autophagy, thereby providing a protective mechanism for metastatic cancer cells against detrimental effects^178^. Promote dormancy: Disseminated tumor cells that fail to establish stable ECM interactions within an unfamiliar microenvironment may enter a state of dormancy, during which they maintain the potential to metastasize when conditions become favorable^179^. Autophagy has been shown to facilitate the survival of these dormant cells *in vivo*^51^. Maintenance of CSCs: CSCs possess the capacity for limitless regeneration, a characteristic that may facilitate tumor metastasis^180^. Autophagy plays a crucial role in sustaining the viability and drug resistance of CSCs, while also preserving a dynamic equilibrium between cancer stem cells and their non-cancerous counterparts^52^. Inhibit senescence: During the initial phases of tumor metastasis, autophagy has the potential to suppress cellular senescence induced by genetic alterations and facilitate cellular proliferation, consequently expediting the progression of tumors^53^.
Inhibit	Tumorigenesis	Remove and degradant damaged cytoplasmic constituents: Autophagy serves as a mechanism for the removal and degradation of impaired cytoplasmic constituents, which encompass protein aggregates and organelles such as mitochondria, endoplasmic reticulum, ribosomes, centrosomes, and lipid droplets^181^-^183^. This process is essential for maintaining cellular quality control. Suppress inflammation: ROS generated during persistent and chronic inflammation contribute to metabolic stress and induce DNA damage^184^. Autophagy serves as a robust anti-inflammatory mechanism that mitigates the activation of inflammatory bodies and modulates type I interferon responses^185^. Suppression of inflammation is a mechanism by which autophagy inhibits tumorigenesis.
	Cancer metastasis	Reduce necrosis: Autophagy facilitates the survival of tumor cells in the face of metabolic stress and hypoxic conditions, which in turn diminishes tumor necrosis and the subsequent infiltration of immune cells, enhancing anti-tumor immunity to restrict the metastasis of tumors^59^. In certain instances, enhanced autophagic flux may trigger cell death mechanisms, including apoptosis, which can impede the process of cancer metastasis^186^. The distinctive mechanism of autophagic cell death contributes to the inhibition of cancer metastasis^60^.

CSC: Cancer stem cell; ECM: Extracellular matrix; EMT: Epithelial-mesenchymal transition; ROS: Reactive oxygen species; TME: Tumor microenvironment.

**Table 2:** Agents and drugs that target the Nrf2-autophagy pathway in cancer.

Cancer types	Agents	Effect on Nrf2-autophagy pathway	Effect on cancer cells	Ref.
NSCLC	Oleifolioside B	Induce Nrf2 dephosphorylation and phosphorylation-Nrf2 nuclear translocation, down-regulate the expression of HO-1	Induce apoptosis and autophagy, result in cell death	139
	Apatinib	Enhance generation of ROS, reduce Nrf2, p62, and Cyclin D1 expression, while promote LC3 B expression. Trigger autophagy and cell death via the ROS/Nrf2/p62 pathway	Inhibit cell proliferation and promote apoptosis and apoptosis, leading to the autophagic and apoptotic cell death	141
	Sodium selenite	Induce a ROS-dependent translocation of Nrf2 to the nucleus, increase ROS-dependent apoptosis and autophagy	Induce apoptosis and autophagy, result in cell death	187
	Isodeoxyelephantopin	Increase expression levels of LC3 B, ATG3, and Beclin1, induce Nrf2 nuclear translocation, and activate downstream target genes *HO-1* and* p62*. Activate protective autophagy through the Nrf2/p62/Keap1 loop	Activate cellular protective autophagy to maintain cancer cell survival	140
Colorectal cancer	L-selenocystine	Induce nuclear translocation of Nrf2 and downstream proteins and autophagy, inhibit Nrf2 pathway (down-regulate Nrf2 and p62 expression, up-regulate Keap1 expression)	Selectively toxic to cells where Nrf2 is continuously activated, causing cell death	116
	Fisetin	Down-regulate Beclin 1 and LC3 expression, up-regulate p62 expression, and inhibit Nrf2 signaling	Inhibit autophagy while induce apoptosis	188
	Δ2-pioglitazone	Increase oxygen and nitric oxide-derived species and decrease glutathione content, activate the Nrf2/Keap1 pathway and autophagy	Reduce cell growth	189
	Metformin	Inhibit the transcriptional activation of Nrf2 and NF-κB, active apoptosis and autophagy in a dose- and time-dependent manner	Reduce cell growth	190
	Lipoic Acid	trigger the depletion of both wild-type and mutant p53 preceded cytotoxicity, stabilize and activate Nrf2, increase p62 level, activate autophagy	Induce cell death	191
	Tributyltin (IV) ferulate	Induce Nrf2-mediated antioxidant response and autophagy	Induce autophagic cell death	192
	Rapamycin in combination with S-allylmercaptocysteine	Activate transcriptional expression of Nrf2 and its downstream gene *NQO1*, down-regulate p62 expression, and activate autophagy	The drug combination can inhibit cell proliferation and enhance their anti-cancer ability	144
	Epigallocatechin-3-gallate in combination with radiation	Significant induce Nrf2 nuclear translocation and up-regulate LC3 expression	Inhibit the growth of cancer cells and increase sensitivity to radiation	193
Oral cancer	Abrus agglutinin	Stimulate ROS production and autophagy activation, up-regulate Nrf2 expression and down-regulate p62 expression	Inhibit the growth of cancer cells	194
	Procaine	Activate the Nrf2 signaling pathway and autophagy	Inhibit growth, differentiation and stemness of cancer cells	152
	Cisplatin	Activate the Nrf2 signaling pathway, which decreases ROS levels	Stimulate autophagy and enrich the cancer stem cells population, leading to cisplatin resistance and tumor recurrence.	35
Prostate cancer	Sulforaphane in combination with Vitamin D	Induce oxidative stress and autophagy, up-regulate the expression of JNK, MAPK, Nrf2 and other proteins	Inhibit the growth of cancer cells	145
	Sinularin	Increase the expression of LC3, Beclin 1 and Nrf2 to induce apoptosis, autophagy and ferroptosis. Increase ROS but reduce glutathione	Inhibit viability, colony formation, migration and invasion of cancer cells	195
Cutaneous tumor	Lycopene	Stimulate antioxidant enzyme activation and Nrf2 translocation, increasing p62 expression, leading to Keap1 degradation.	Reduce the incidence and diversity of skin tumors during the promotion period and the tumorigenic capacity of normal skin cells	100
Breast cancer	Naringenin	Inhibit autophagy in breast cancer cells via FKBP4/NR3C1/NRF2 signaling pathway	Inhibit autophagy and proliferation of cancer cells, and promote the differentiation and maturation of dendritic cells	196
	Atractylenolide-III	Suppress inflammation and oxidative stress through the Nrf2/ARE pathway, induce Nrf2 expression through the autophagic degradation of Keap1	Reduce tumor volume and multiplicity, prolong tumor latency, and reverse weight loss	197
	Mitoquinone	Induce ATG7-mediated autophagy. Increase ROS level and trigger the Keap1-Nrf2 antioxidant response	Selectively kill breast cancer cells over healthy mammary epithelial cells	198
	Isoaaptamine	Induce autophagy through the Nrf2/p62 signaling	Inhibit proliferation and clone formation of cancer cells	199
Renal cancer	Cabozantinib in combination with Honokiol	Decrease the expression of p62 and Nrf2, increase ROS production, and active autophagy	Inhibit the growth of cancer cells and promotes apoptosis and autophagy-mediated cell death	114
Esophageal squamous cell carcinoma	Sulforaphane in combination with CQ	Induce cellular protective autophagy by activating Nrf2	CQ can neutralize the activation of autophagy, and improve the anti-cancer activity of Sulforaphane	115
	Polygalacin D	Induce the accumulation of ROS and the activation of autophagy, reduce Nrf2 expression in a dose- and time-dependent manner	Inhibit the proliferation, migration and invasion of cancer cells, repress tumor growth and lung metastasis	200
Head and neck cancer	RITA in combination with 3-MA	Autophagy-related proteins and p62 are highly expressed in RITA-resistant cancer cells, accompanied by activation of the Keap1-Nrf2 pathway. 3-MA inhibits autophagy and the antioxidant system	RITA induces cancer cell apoptosis. 3-MA sensitizes RITA-resistant cancer cells.	201
Bladder cancer	Jaspine B derivative C-2	Stimulate the Nrf2 pathway by activating JNK to trigger autophagy and up-regulate p62 expression	Inhibit the growth of cancer cells	142
Gastric cancer	Jaspine B derivative C-2	Stimulate cell-protective autophagy via the JNK/ERK/Beclin 1/Nrf2/p62 pathway	Inhibit the growth of cancer cells	121
	Diclofenac in combination with cisplatin	Increase ROS level, inhibit Nrf2 activity, induce autophagy	Induce autophagic cell death, reverse cisplatin resistance	202
Pancreatic cancer	Apigenin	Activate the Nrf2-p62 pathway and up-regulate antioxidant response	Inhibit the growth of cancer cells	203
	Resveratrol	Induce ROS accumulation and activate Nrf2 signaling. Trigger apoptosis and autophagy	Inhibit the proliferation of cancer cells. Improve the sensitivity of cancer cells to gemcitabine	204
HCC	Caryophyllene oxide	Promote ROS production and lipid peroxidation. Inhibit Nrf2 and HO-1 expression. Activate ferritinophagy	Inhibit the growth of cancer cells	205
	Sarmentosin	Activate apoptosis and autophagy, increase Nrf2 nuclear translocation and up-regulate Nrf2 target gene expression	Inhibit the growth of cancer cells	206
Cervical cancer	Sulforaphane	Increase expression of Nrf2 and p62, activate autophagy	Inhibit the growth of cancer cells	138
Glioblastoma	FTY720	Inhibit the expression of Nrf2, HO-1 and NQO-1, trigger autophagy	Inhibit the migration and invasion of cancer cells. Sensitize cancer cells to temozolomide	207
	Temozolomide	Induce autophagy and increase Nrf2 expression	Inhibit viability of cancer cells	150
Leukemia	BIX-01294	Activate the PERK/NRF2 pathway and increase HO-1 expression, inhibit ROS accumulation, induce autophagy	Inhibit the growth of cancer cells	208
Ovarian cancer	Apatinib	Inhibit glutathione to generate ROS through the Nrf2/HO-1 pathway. Activate ROS-dependent autophagy	Inhibit the growth and migration of cancer cells in a dose- and time-dependent manner	147
Thyroid cancer	Pinelliae rhizome	Inhibit Nrf2 expression in a dose-dependent manner, activate autophagy	Inhibit the growth and proliferation of cancer cells	209

3-MA: 3-methyladenine; CQ: Chloroquine; HCC: Hepatocellular carcinoma; NSCLC: non-small cell lung cancer; RITA: Reactivation of p53 and induction of tumor cell apoptosis.

**Table 3:** Nrf2-p62 and other autophagy-related proteins in cancer monitoring

Proteins	Cancer types	Role in cancer monitoring	Ref.
Nrf2	Several kinds of solid and hematologic tumors	Persistent Nrf2 expression and function in healthy cells increase the likelihood of cancer development. Increased Nrf2 expression and activity are associated with adverse outcomes in the advancement of tumors. Nrf2 activity is sustained through the autophagy signaling pathway, with p62 serving as a pivotal point for interaction and transposition between autophagy and the Nrf2-Keap1 pathway. Nrf2 signaling modulates the responsiveness of cancer cells to chemotherapy drugs.	153-156, 208, 210-212
p62	Several kinds of solid and hematologic tumors	Marker of autophagy signaling activation, promoting Nrf2 activation. p62 is abnormally accumulated and up-regulated in a variety of cancers, which can induce the occurrence of cancer. High p62 expression is often associated with poor cancer prognosis.	16, 157-159, 211
LC3 A/B	Several kinds of solid and hematologic tumors	LC3 A expression is up-regulated in HCC; associated with serum alpha-fetoprotein and poor tumor differentiation. "Stone-like" LC3 A expression is an independent predictor of HCC prognosis. High expression of LC3 B is closely associated with larger tumors, later tumor staging, and poorer recurrence-free survival and overall survival. Will serve as a potential molecular marker to guide tumor staging and surgical treatment options.	16, 17, 160, 161, 213, 214
GABARAP/ GABARAPL1/2	Breast cancer	GABARAP inhibits tumor proliferation, migration, and invasion while inducing EMT. GABARAP is inversely associated with tumor size and TNM stage. Patients with low GABARAP levels have a poorer prognosis. GABARAP can be used as a diagnostic marker and therapeutic target for breast cancer.	215
	Colorectal cancer	GABARAP, GABARAPL1/2 are down-regulated in tumor tissues, which can be used as potential biomarkers as well as putative targets in CRC diagnosis and therapy.	216
	AML	Low expression of GABARAPL1/2 is associated with immature myeloid leukemia phenotype	217
	Thyroid cancer	Increased expression of GABARAP in tumors suggests that it plays a role in the early stages of thyroid carcinogenesis and may serve as a potential diagnostic marker.	218
UVRAG	Colorectal cancer	UVRAG is related to tumor staging, differentiation, and distal metastasis, and enhances tumor migration and drug resistance by up-regulating SP1 and PD-1 expression, leading to poor colorectal cancer prognosis.	162
	HCC	UVRAG S522 phosphorylation levels are associated with poor prognosis in HCC patients	163
ULK1	HCC	ULK1 expression was negatively correlated with PFS. ULK1 is a prognostic factor in HCC, and combined with LC3 B can improve the prognostic assessment.	164
	Breast cancer	Low ULK1 expression is associated with breast cancer progression and decreased autophagy. ULK1 can be used as a prognostic biomarker in breast cancer patients.	165
	Nasopharyngeal carcinoma	High ULK1 expression is closely associated with aggressiveness and treatment resistance in patients, and is negatively associated with DSS. It can be used to predict treatment response and patient survival outcomes of nasopharyngeal carcinoma.	166
Beclin 1	NSCLC	Low Beclin 1 expression in NSCLC is closely related to staging, lymph node metastasis, and tumor differentiation. It can be used for active follow-up and timely treatment of HCC patients.	167
	Gastric cancer	Low Beclin 1 expression is positively correlated with lymph node metastasis, TNM staging, dedifferentiation, and poor prognosis. It can be used as a potential marker for the occurrence, aggressiveness and prognosis of gastric cancer, and may become a novel therapeutic target.	168
	ICC	Low Beclin 1 expression is associated with lymph node metastasis, poor overall survival, and poor overall survival. Beclin 1 is related to the progression and metastasis of ICC and may serve as a new marker for ICC prognosis.	169
	Lung adenocarcinoma	Beclin 1 expression is up-regulated in bone metastases and may be used as a prognostic marker for bone metastasis.	16

AML: Acute myelogenous leukemia; EMT: Epithelial-mesenchymal transition; HCC: Hepatocellular carcinoma; ICC: Intrahepatic cholangiocellular carcinoma; DSS: Disease-specific survival; NSCLC: Non-small cell lung cancer; PFS: Progression free survival; TNM: Tumor node metastasis.

**Table 4:** Clinical trials of Nrf2 and autophagy signaling in cancer.

Target	Medication pattern	Cancer types	Phase	Overview of the clinical trials	ClinicalTrials ID
Nrf2 or Nrf2/Keap1	Medication alone	HNSC	Phase 0	To test the safety of Pyrimethamine as an inhibitor of Nrf2 in HPV-negative, locally advanced HNSCC	NCT05678348
			Phase 0	To determine the oral bioavailability of Sulforaphane in the commercially available dietary supplement, Avmacol®, and upregulation level of Nrf2 target gene transcripts in tobacco-related HNSCC	NCT03182959
		Solid Tumors	Phase II	To test the inhibitory effect of glutaminase inhibitor Telaglenastat hydrochloride on the growth of Keap1/Nrf2 aberrant tumors	NCT03872427
		NSCLC	Phase I	To test the safety and tolerability of MGY825 in advanced NSCLC harboring Nrf2/Keap1/CUL3 mutations	NCT05275868
			Phase II	To test the effect of TORC 1/2 inhibitor Sapanisertib in relapsed/refractory Nrf2-mutated NSCLC	NCT02417701
		Advanced solid tumors	Phase I	To test the safety, tolerability, pharmacology, and anti-tumor activity of the Keap1 activator VVD-130037 in advanced solid tumors	NCT05954312
		Heavy Smokers	Phase II	To test the effect of the broccoli seed and sprout extract, Avmacol ES, on the cancer-causing substances of tobacco in heavy smokers	NCT05121051
	Medication combination	NSCLC	Phase II	To test the effect of the glutaminase inhibitor Telaglenastat with Pembrolizumab and chemotherapy versus placebo with Pembrolizumab and chemotherapy in first-line, metastatic Keap1/Nrf2-mutated NSCLC	NCT04265534
		AML	Phase II	To test the anti-tumor effect of Pevonedistat with Azacitidine in adult relapsed or refractory AML by calculate the expression of Nrf2 target genes NQO1 and SLC7A11	NCT03745352
Autophagy	Medication alone	Breast cancer	Phase II	To investigate the inhibition of autophagy caused by HCQ in breast cancer	NCT01292408
			Phase II	To test the effect of chloroquine in breast cancer	NCT02333890
		Myeloma	Phase 0	To test the induction of autophagy by Bortezomib in myeloma	NCT01594242
			Phase 0	To test the effect of HCQ in stage III or stage IV melanoma	NCT00962845
		SCLC	Phase I	To test the efficiency of chloroquine as an anti-autophagy drug in stage IV SCLC	NCT00969306
		Prostate cancer	Phase 0	To test the biological effect of HCQ in prostate cancer	NCT02421575
			Phase II	To test the effect of HCQ in treating patients with previously treated prostate cancer	NCT00726596
		Renal cell carcinoma	Phase I	To test the efficiency of pre-operative HCQ in primary renal cell carcinoma	NCT01144169
	Medication combination	Advanced cancer	Phase I	To measure the highest tolerable dose and safety of MLN9708 and Vorinostat combination to target autophagy in advanced p53 mutant cancer	NCT02042989
			Phase I	To test the highest tolerable dose and safety of Sirolimus or Vorinostat in combination with HCQ to with advanced cancer	NCT01266057
			Phase I	To evaluate the maximum tolerated dose, safety and anti-tumor activity of HCQ in combination with Vorinostat in advanced solid tumors	NCT01023737
			Phase I	To test the safety and efficacy of HCQ in combination with Temsirolimus in refractory solid tumors	NCT00909831
			Phase I	To test the side effects and best dose of Sunitinib malate in combination with HCQ in advanced solid tumors that have not responded to chemotherapy	NCT00813423
			Phase I	To test the efficiency of Sorafenib in refractory or relapsed solid tumors, and mitigate its toxicity by combining with HCQ	NCT01634893
			Phase I	To test the maximum tolerated dose and side effects of AKT inhibitor MK2206 in combination with HCQ in advanced solid tumors	NCT01480154
			Phase I/II	To test the safety and efficiency of Cobimetinib, Atezolizumab and HCQ combination in KRAS-mutated advanced cancer	NCT04214418
			Phase II	To test the efficacy and safety of Chloroquine plus radiation in brain metastases from solid tumors.	NCT01894633
			Phase I/II	To test the safety and efficacy of combination of autophagy selective therapeutics in advanced solid tumors	NCT05036226
		Breast cancer	Phase 0	To test the efficiency of quadruple therapy Quercetin, Zinc, Metformin, and EGCG in early, metastatic breast cancer and triple-negative breast cancer	NCT05680662
			Phase I	To test the efficiency of HCQ in combination with Abemaciclib and endocrine therapy in HR^+^/HER 2-advanced breast cancer	NCT04316169
			Phase I/II	To test the safety and anti-tumor efficacy of CDK4/6 inhibitor Palbociclib in combination with HCQ in HR^+^HER2-breast cancer	NCT05953350
			Phase I/II	To test the safety and anti-tumor efficacy of Palbociclib plus Letrozole in combination with HCQ in ER^+^HER2-breast cancer	NCT03774472
			Phase I/II	To test the anti-tumor activity of Ixabepilone in combination with HCQ in metastatic breast cancer	NCT00765765
			Phase II	To test the efficiency of ABemacicliB or Abemaciclib and HCQ to target minimal residual disease in breast cancer	NCT04523857
			Phase II	To test the efficiency of Avelumab or HCQ in combination with Palbociclib in dormant breast cancer	NCT04841148
		Cholangiocar-cinoma	Phase II	To test the efficiency of ABC294640 (Yeliva ®) Alone and in combination with HCQ in advanced cholangiocarcinoma	NCT03377179
			Phase II	To test the efficiency of MEK inhibitor Trametinib in combination with HCQ in KRAS mutation refractory bile tract carcinoma	NCT04566133
		HCC	Phase I/II	To test the safety and efficacy of the combination of HCQ with trans-arterial chemoembolization to treat intermediate stage HCC	NCT05842174
			Phase II	To investigate Sorafenib-induced autophagy using HCQ in HCC	NCT03037437
		Prostate cancer	Phase I	To test the side effects and best dose of Metformin hydrochloride in combination with Enzalutamide to overcome autophagy resistance in castration resistant prostate cancer	NCT02339168
			Phase I	To test the efficiency of ADI-PEG 20 plus docetaxel in prostate cancer and NSCLC	NCT01497925
			Phase II	To test the effect of docetaxel in combination with HCQ in metastatic prostate cancer	NCT00786682
			Phase II	To test the efficiency of ABT-263/Abiraterone or ABT-263/Abiraterone in combination with HCQ in metastatic castrate refractory prostate cancer	NCT01828476
		Gastric cancer	Phase II	To test the safety and efficacy of Ulixertinib in combination with HC1 in RAS, ERK and MEK mutated gastric cancer	NCT05221320
		Colorectal cancer	Phase Ib	To test the efficiency of hepatic chemoembolization plus Axitinib and HCQ in liver-dominant metastatic colorectal cancer	NCT04873895
			Phase I/II	To test the effect of HCQ in combination with Folfox plus Bevacizumab in colorectal cancer	NCT01206530
			Phase I/II	To test the efficiency of Vorinostat plus HCQ versus Regorafenib in refractory metastatic colorectal cancer	NCT02316340
			Phase II	To test the efficiency of HCQ in combination with Encorafenib and Cetuximab or Panitumumab in metastatic BRAF-mutated colorectal cancer	NCT05576896
		Pancreatic cancer	Phase II	To test the anti-tumor efficiency of ERK inhibitor LY3214996 with and without HCQ in advanced pancreatic cancer	NCT04386057
			Phase I/II	To test the toxicity of Gemcitabine/Abraxane in combination with HCQ in pancreatic cancer	NCT01506973
			Phase I/II	To test the efficiency of pre-operative Gemcitabine and Nab Paclitacel in combination with HCQ in pancreatic cancer	NCT01978184
			Phase II	To test the efficiency of pre-operative Gemcitabine, Nab-Paclitaxel, and HCQ with Avelumab in pancreatic cancer	NCT03344172
			Phase II	To test the efficiency of short course radiation therapy with proton or photon beam Capecitabine and HCQ in respectable pancreatic cancer	NCT01494155
			Phase II	To test the efficiency of Paricalcitol and HCQ in combination with Gemcitabine and Nab-paclitaxel in advanced pancreatic cancer	NCT04524702
		Melanoma	Phase I/II	To test the safety and tolerability of the combination of HCQ and MEK inhibitor Trametinib in metastatic neuroblastoma RAS melanoma	NCT03979651
			Phase I/II	To test the efficiency of Dabrafenib, Trametinib and HCQ in advanced BRAF mutant melanoma	NCT02257424
			Phase II	To test the efficiency of Dabrafenib and Trametinib in combination with HCQ in advanced BRAF V600E/K melanoma	NCT04527549
		NSCLC	Phase II	To test the effect of Paclitaxel and Carboplatinone in combination with HCQ in advanced/recurrent NSCLC	NCT01649947
		Myeloma	Phase 0	To test the efficiency of Cyclophosphamide and pulse Dexamethasone with Rapamycin or HCQ in relapsed or refractory multiple myeloma	NCT01396200
			Phase I	To test the efficiency of Carfilzomib in combination with HCQ in relapsed/refractory multiple myeloma	NCT04163107
			Phase I/II	To test the side effects, best dose and efficiency of Bortezomib in combination with HCQ in relapsed or refractory multiple myeloma	NCT00568880
			Phase II	To test the efficiency of Chloroquine in combination with Velcade and Cyclophosphamide in relapsed and refractory myeloma	NCT01438177
		Glioblastoma	Phase I	To test the maximum tolerated dose of chloroquine in combination with radiotherapy with Temozolomide in glioblastoma	NCT02378532
			Phase I/II	To test the efficiency of HCQ, radiation, and Temozolomide in glioblastoma multiforme	NCT00486603
			Phase I/II	To test the efficiency of Dabrafenib, Trametinib, and HCQ for BRAF V600E-mutant or Trametinib and HCQ for BRAF fusion/duplication positive or NF1-associated recurrent or progressive gliomas	NCT04201457
		Osteosarcoma	Phase I/II	To test the efficiency of Gemcitabine and Docetaxel in combination with HCQ in recurrent osteosarcoma	NCT03598595
		Renal cell carcinoma	Phase I/II	To test the efficiency of RAD001 in combination with HCQ in previously treated renal cell carcinoma	NCT01510119
		AML	Phase I	To test the efficiency of Mitoxantrone and Etoposide in combination with HCQ in relapsed AML	NCT02631252

AML: Acute myelogenous leukemia; HCC: Hepatocellular carcinoma; HCQ: Hydroxychloroquine; HNSCC: Head and neck squamous cell carcinoma; NSCLC: Non-small cell lung cancer; SCLC: Small cell lung cancer.

## Abbreviations

3-MA: 3-methyladenine
AML: acute myelogenous leukemia
ARE: antioxidant response element
ATG: autophagy-related gene
CAFs: cancer-associated fibroblasts
CQ: chloroquine
Cul3: Cullin 3
CSCs: cancer stem cells
DSS: disease-specific survival
EMT: Epithelial-mesenchymal transition
ESI: isodeoxyelephantopin
Fn OMVs: fusobacterium nucleatum outer membrane vesicles
GABARAP: gamma-aminobutyric acid type A receptor-associated protein
GCL: glutamate cysteine ligase
HCC: hepatocellular carcinoma
HCQ: hydroxychloroquine
HNSCC: head and neck squamous cell carcinoma
ICC: intrahepatic cholangiocellular carcinoma
Keap1: kelch-like ECH-associated protein 1
LC3: microtubule-associated protein 1 light chain 3
Maf: muscle tendon membrane fibrosarcoma
mTOR: mammalian TOR
Neh: nrf2 ECH homology domain
NQO1: NAD(P)H quinone dehydrogenase 1
Nrf2: nuclear factor (erythroid derived-2)-like 2
NSCLC: non-small cell lung cancer
OSCC: oral squamous cell carcinoma
PE: phosphatidylethanolamine
PFS: progression free survival
PI3K: phosphatidylinositol 3-kinase
PINK1: PTEN induced putative kinase 1
RITA: reactivation of p53 and induction of tumor cell apoptosis
ROS: reactive oxygen species
SAMC: S-allylmercaptocysteine
SCLC: small cell lung cancer
TME: the tumor microenvironment
TNM: tumor node metastasis
TOR: target of rapamycin
ULK1/2: serine/threonine UNC-51-like kinases 1/2
UVRAG: UV radiation resistance-associated gene protein

## References

[1] Shan C, Chen X, Cai H, Hao X, Li J, Zhang Y (2021). The Emerging Roles of Autophagy-Related MicroRNAs in Cancer. Int J Biol Sci.

[2] Devis-Jauregui L, Eritja N, Davis ML, Matias-Guiu X, Llobet-Navas D (2021). Autophagy in the physiological endometrium and cancer. Autophagy.

[3] Yamamoto H, Zhang S, Mizushima N (2023). Autophagy genes in biology and disease. Nat Rev Genet.

[4] Li J, Zhan H, Ren Y, Feng M, Wang Q, Jiao Q (2023). Sirtuin 4 activates autophagy and inhibits tumorigenesis by upregulating the p53 signaling pathway. Cell Death Differ.

[5] Liu Y, Liu Y, He Y, Zhang N, Zhang S, Li Y (2023). Hypoxia-Induced FUS-circTBC1D14 Stress Granules Promote Autophagy in TNBC. Adv Sci (Weinh).

[6] Wang X, Xu F, Kou H, Zheng Y, Yang J, Xu Z (2023). Stromal cell-derived small extracellular vesicles enhance radioresistance of prostate cancer cells via interleukin-8-induced autophagy. J Extracell Vesicles.

[7] Ren Y, Wang R, Weng S, Xu H, Zhang Y, Chen S (2023). Multifaceted role of redox pattern in the tumor immune microenvironment regarding autophagy and apoptosis. Mol Cancer.

[8] Liu S, Pi J, Zhang Q (2022). Signal amplification in the KEAP1-NRF2-ARE antioxidant response pathway. Redox Biol.

[9] Crisman E, Duarte P, Dauden E, Cuadrado A, Rodriguez-Franco MI, Lopez MG (2023). KEAP1-NRF2 protein-protein interaction inhibitors: Design, pharmacological properties and therapeutic potential. Med Res Rev.

[10] Deng Z, Lim J, Wang Q, Purtell K, Wu S, Palomo GM (2020). ALS-FTLD-linked mutations of SQSTM1/p62 disrupt selective autophagy and NFE2L2/NRF2 anti-oxidative stress pathway. Autophagy.

[11] Lee DH, Park JS, Lee YS, Han J, Lee DK, Kwon SW (2020). SQSTM1/p62 activates NFE2L2/NRF2 via ULK1-mediated autophagic KEAP1 degradation and protects mouse liver from lipotoxicity. Autophagy.

[12] Su H, Yang F, Fu R, Li X, French R, Mose E (2021). Cancer cells escape autophagy inhibition via NRF2-induced macropinocytosis. Cancer Cell.

[13] Shi Q, Jin X, Zhang P, Li Q, Lv Z, Ding Y (2022). SPOP mutations promote p62/SQSTM1-dependent autophagy and Nrf2 activation in prostate cancer. Cell Death Differ.

[14] Levine B, Kroemer G (2019). Biological Functions of Autophagy Genes: A Disease Perspective. Cell.

[15] Brun S, Bestion E, Raymond E, Bassissi F, Jilkova ZM, Mezouar S (2022). GNS561, a clinical-stage PPT1 inhibitor, is efficient against hepatocellular carcinoma via modulation of lysosomal functions. Autophagy.

[16] Li D, He C, Ye F, Ye E, He H, Chen G (2021). p62 Overexpression Promotes Bone Metastasis of Lung Adenocarcinoma out of LC3-Dependent Autophagy. Front Oncol.

[17] Shen N, Wang L, Wu J, Chen X, Hu F, Su Y (2023). Meta-analysis of the autophagy-associated protein LC3 as a prognostic marker in colorectal cancer. Exp Ther Med.

[18] Mauthe M, Orhon I, Rocchi C, Zhou X, Luhr M, Hijlkema KJ (2018). Chloroquine inhibits autophagic flux by decreasing autophagosome-lysosome fusion. Autophagy.

[19] Lamark T, Johansen T (2021). Mechanisms of Selective Autophagy. Annu Rev Cell Dev Biol.

[20] Vara-Perez M, Felipe-Abrio B, Agostinis P (2019). Mitophagy in Cancer: A Tale of Adaptation. Cells.

[21] Picca A, Faitg J, Auwerx J, Ferrucci L, D'Amico D (2023). Mitophagy in human health, ageing and disease. Nat Metab.

[22] Li X, He S, Ma B (2020). Autophagy and autophagy-related proteins in cancer. Mol Cancer.

[23] Ames K, Kaur I, Shi Y, Tong MM, Sinclair T, Hemmati S (2023). PI3-kinase deletion promotes myelodysplasia by dysregulating autophagy in hematopoietic stem cells. Sci Adv.

[24] Lascaux P, Hoslett G, Tribble S, Trugenberger C, Anticevic I, Otten C (2024). TEX264 drives selective autophagy of DNA lesions to promote DNA repair and cell survival. Cell.

[25] Liu H, Zhen C, Xie J, Luo Z, Zeng L, Zhao G (2024). TFAM is an autophagy receptor that limits inflammation by binding to cytoplasmic mitochondrial DNA. Nat Cell Biol.

[26] Chao X, Wang S, Fulte S, Ma X, Ahamed F, Cui W (2022). Hepatocytic p62 suppresses ductular reaction and tumorigenesis in mouse livers with mTORC1 activation and defective autophagy. J Hepatol.

[27] Lin Z, Yang S, Qiu Q, Cui G, Zhang Y, Yao M (2024). Hypoxia-induced cysteine metabolism reprogramming is crucial for the tumorigenesis of colorectal cancer. Redox Biol.

[28] Jia M, Yue X, Sun W, Zhou Q, Chang C, Gong W (2023). ULK1-mediated metabolic reprogramming regulates Vps34 lipid kinase activity by its lactylation. Sci Adv.

[29] Li W, Zhou C, Yu L, Hou Z, Liu H, Kong L (2024). Tumor-derived lactate promotes resistance to bevacizumab treatment by facilitating autophagy enhancer protein RUBCNL expression through histone H3 lysine 18 lactylation (H3K18la) in colorectal cancer. Autophagy.

[30] Wijshake T, Zou Z, Chen B, Zhong L, Xiao G, Xie Y (2021). Tumor-suppressor function of Beclin 1 in breast cancer cells requires E-cadherin. Proc Natl Acad Sci U S A.

[31] Tan P, He L, Xing C, Mao J, Yu X, Zhu M (2019). Myeloid loss of Beclin 1 promotes PD-L1hi precursor B cell lymphoma development. J Clin Invest.

[32] Frangez Z, Seyed Jafari SM, Hunger RE, Simon HU (2020). Loss of Concurrent Regulation of the Expression of BIF-1, BAX, and Beclin-1 in Primary and Metastatic Melanoma. Biochemistry (Mosc).

[33] Li X, Yang KB, Chen W, Mai J, Wu XQ, Sun T (2021). CUL3 (cullin 3)-mediated ubiquitination and degradation of BECN1 (beclin 1) inhibit autophagy and promote tumor progression. Autophagy.

[34] Keulers TG, Koch A, van Gisbergen MW, Barbeau LMO, Zonneveld MI, de Jong MC (2022). ATG12 deficiency results in intracellular glutamine depletion, abrogation of tumor hypoxia and a favorable prognosis in cancer. Autophagy.

[35] Praharaj PP, Singh A, Patra S, Bhutia SK (2023). Co-targeting autophagy and NRF2 signaling triggers mitochondrial superoxide to sensitize oral cancer stem cells for cisplatin-induced apoptosis. Free Radic Biol Med.

[36] Xu Z, Han X, Ou D, Liu T, Li Z, Jiang G (2020). Targeting PI3K/AKT/mTOR-mediated autophagy for tumor therapy. Appl Microbiol Biotechnol.

[37] White E, Karp C, Strohecker AM, Guo Y, Mathew R (2010). Role of autophagy in suppression of inflammation and cancer. Curr Opin Cell Biol.

[38] Mantovani A, Allavena P, Sica A, Balkwill F (2008). Cancer-related inflammation. Nature.

[39] Debnath J, Gammoh N, Ryan KM (2023). Autophagy and autophagy-related pathways in cancer. Nat Rev Mol Cell Biol.

[40] Mukhopadhyay S, Encarnacion-Rosado J, Lin EY, Sohn ASW, Zhang H, Mancias JD (2023). Autophagy supports mitochondrial metabolism through the regulation of iron homeostasis in pancreatic cancer. Sci Adv.

[41] Chen W, Zhao H, Li Y (2023). Mitochondrial dynamics in health and disease: mechanisms and potential targets. Signal Transduct Target Ther.

[42] Karsli-Uzunbas G, Guo JY, Price S, Teng X, Laddha SV, Khor S (2014). Autophagy is required for glucose homeostasis and lung tumor maintenance. Cancer Discov.

[43] Yang A, Herter-Sprie G, Zhang H, Lin EY, Biancur D, Wang X (2018). Autophagy Sustains Pancreatic Cancer Growth through Both Cell-Autonomous and Nonautonomous Mechanisms. Cancer Discov.

[44] Sousa CM, Biancur DE, Wang X, Halbrook CJ, Sherman MH, Zhang L (2016). Pancreatic stellate cells support tumour metabolism through autophagic alanine secretion. Nature.

[45] Martinez-Outschoorn UE, Lisanti MP, Sotgia F (2014). Catabolic cancer-associated fibroblasts transfer energy and biomass to anabolic cancer cells, fueling tumor growth. Semin Cancer Biol.

[46] New J, Arnold L, Ananth M, Alvi S, Thornton M, Werner L (2017). Secretory Autophagy in Cancer-Associated Fibroblasts Promotes Head and Neck Cancer Progression and Offers a Novel Therapeutic Target. Cancer Res.

[47] Li X, Lee Y, Kang Y, Dai B, Perez MR, Pratt M (2019). Hypoxia-induced autophagy of stellate cells inhibits expression and secretion of lumican into microenvironment of pancreatic ductal adenocarcinoma. Cell Death Differ.

[48] Chang CP, Su YC, Hu CW, Lei HY (2013). TLR2-dependent selective autophagy regulates NF-kappaB lysosomal degradation in hepatoma-derived M2 macrophage differentiation. Cell Death Differ.

[49] Alissafi T, Hatzioannou A, Mintzas K, Barouni RM, Banos A, Sormendi S (2018). Autophagy orchestrates the regulatory program of tumor-associated myeloid-derived suppressor cells. J Clin Invest.

[50] Zanotelli MR, Zhang J, Reinhart-King CA (2021). Mechanoresponsive metabolism in cancer cell migration and metastasis. Cell Metab.

[51] Vera-Ramirez L (2020). Cell-intrinsic survival signals. The role of autophagy in metastatic dissemination and tumor cell dormancy. Semin Cancer Biol.

[52] Nazio F, Bordi M, Cianfanelli V, Locatelli F, Cecconi F (2019). Autophagy and cancer stem cells: molecular mechanisms and therapeutic applications. Cell Death Differ.

[53] Liu H, He Z, von Rutte T, Yousefi S, Hunger RE, Simon HU (2013). Down-regulation of autophagy-related protein 5 (ATG5) contributes to the pathogenesis of early-stage cutaneous melanoma. Sci Transl Med.

[54] Xu H, Wang J, Al-Nusaif M, Ma H, Le W (2024). CCL2 promotes metastasis and epithelial-mesenchymal transition of non-small cell lung cancer via PI3K/Akt/mTOR and autophagy pathways. Cell Prolif.

[55] Chen G, Gao C, Jiang S, Cai Q, Li R, Sun Q (2024). Fusobacterium nucleatum outer membrane vesicles activate autophagy to promote oral cancer metastasis. J Adv Res.

[56] Zamora A, Alves M, Chollet C, Therville N, Fougeray T, Tatin F (2019). Paclitaxel induces lymphatic endothelial cells autophagy to promote metastasis. Cell Death Dis.

[57] La Belle Flynn A, Calhoun BC, Sharma A, Chang JC, Almasan A, Schiemann WP (2019). Autophagy inhibition elicits emergence from metastatic dormancy by inducing and stabilizing Pfkfb3 expression. Nat Commun.

[58] Aqbi HF, Tyutyunyk-Massey L, Keim RC, Butler SE, Thekkudan T, Joshi S (2018). Autophagy-deficient breast cancer shows early tumor recurrence and escape from dormancy. Oncotarget.

[59] Degenhardt K, Mathew R, Beaudoin B, Bray K, Anderson D, Chen G (2006). Autophagy promotes tumor cell survival and restricts necrosis, inflammation, and tumorigenesis. Cancer Cell.

[60] Liu W, Zhao Y, Wang G, Feng S, Ge X, Ye W (2022). TRIM22 inhibits osteosarcoma progression through destabilizing NRF2 and thus activation of ROS/AMPK/mTOR/autophagy signaling. Redox Biol.

[61] Sies H, Jones DP (2020). Reactive oxygen species (ROS) as pleiotropic physiological signalling agents. Nat Rev Mol Cell Biol.

[62] Karan A, Bhakkiyalakshmi E, Jayasuriya R, Sarada DVL, Ramkumar KM (2020). The pivotal role of nuclear factor erythroid 2-related factor 2 in diabetes-induced endothelial dysfunction. Pharmacol Res.

[63] Tian W, Rojo de la Vega M, Schmidlin CJ, Ooi A, Zhang DD (2018). Kelch-like ECH-associated protein 1 (KEAP1) differentially regulates nuclear factor erythroid-2-related factors 1 and 2 (NRF1 and NRF2). J Biol Chem.

[64] Al-Mubarak BR, Bell KFS, Chowdhry S, Meakin PJ, Baxter PS, McKay S (2021). Non-canonical Keap1-independent activation of Nrf2 in astrocytes by mild oxidative stress. Redox Biol.

[65] Fu W, Xiao Z, Chen Y, Pei J, Sun Y, Zhang Z (2023). Molecular integrative study on interaction domains of nuclear factor erythroid 2-related factor 2 with sirtuin 6. Biochimie.

[66] Chen Y, Liu K, Zhang J, Hai Y, Wang P, Wang H (2020). c-Jun NH(2) -Terminal Protein Kinase Phosphorylates the Nrf2-ECH Homology 6 Domain of Nuclear Factor Erythroid 2-Related Factor 2 and Downregulates Cytoprotective Genes in Acetaminophen-Induced Liver Injury in Mice. Hepatology.

[67] Wang H, Liu K, Geng M, Gao P, Wu X, Hai Y (2013). RXRalpha inhibits the NRF2-ARE signaling pathway through a direct interaction with the Neh7 domain of NRF2. Cancer Res.

[68] Zhang W, Feng C, Jiang H (2021). Novel target for treating Alzheimer's Diseases: Crosstalk between the Nrf2 pathway and autophagy. Ageing Res Rev.

[69] Wang X, Zhou T, Yang X, Cao X, Jin G, Zhang P (2023). DDRGK1 Enhances Osteosarcoma Chemoresistance via Inhibiting KEAP1-Mediated NRF2 Ubiquitination. Adv Sci (Weinh).

[70] Liu S, Pi J, Zhang Q (2021). Mathematical modeling reveals quantitative properties of KEAP1-NRF2 signaling. Redox Biol.

[71] Niture SK, Jain AK, Jaiswal AK (2009). Antioxidant-induced modification of INrf2 cysteine 151 and PKC-delta-mediated phosphorylation of Nrf2 serine 40 are both required for stabilization and nuclear translocation of Nrf2 and increased drug resistance. J Cell Sci.

[72] Ishii T, Warabi E, Mann GE (2022). Mechanisms underlying Nrf2 nuclear translocation by non-lethal levels of hydrogen peroxide: p38 MAPK-dependent neutral sphingomyelinase2 membrane trafficking and ceramide/PKCzeta/CK2 signaling. Free Radic Biol Med.

[73] Tao T, Wang J, Wang X, Wang Y, Mao H, Liu X (2019). The PERK/Nrf2 pathway mediates endoplasmic reticulum stress-induced injury by upregulating endoplasmic reticulophagy in H9c2 cardiomyoblasts. Life Sci.

[74] Xu C, Yuan X, Pan Z, Shen G, Kim JH, Yu S (2006). Mechanism of action of isothiocyanates: the induction of ARE-regulated genes is associated with activation of ERK and JNK and the phosphorylation and nuclear translocation of Nrf2. Mol Cancer Ther.

[75] Wang Z, Yao M, Jiang L, Wang L, Yang Y, Wang Q (2022). Dexmedetomidine attenuates myocardial ischemia/reperfusion-induced ferroptosis via AMPK/GSK-3beta/Nrf2 axis. Biomed Pharmacother.

[76] Miao W, Hu L, Scrivens PJ, Batist G (2005). Transcriptional regulation of NF-E2 p45-related factor (NRF2) expression by the aryl hydrocarbon receptor-xenobiotic response element signaling pathway: direct cross-talk between phase I and II drug-metabolizing enzymes. J Biol Chem.

[77] Kubli SP, Bassi C, Roux C, Wakeham A, Gobl C, Zhou W (2019). AhR controls redox homeostasis and shapes the tumor microenvironment in BRCA1-associated breast cancer. Proc Natl Acad Sci U S A.

[78] Tang YC, Hsiao JR, Jiang SS, Chang JY, Chu PY, Liu KJ (2021). c-MYC-directed NRF2 drives malignant progression of head and neck cancer via glucose-6-phosphate dehydrogenase and transketolase activation. Theranostics.

[79] Zhao L, Qi Y, Xu L, Tao X, Han X, Yin L (2018). MicroRNA-140-5p aggravates doxorubicin-induced cardiotoxicity by promoting myocardial oxidative stress via targeting Nrf2 and Sirt2. Redox Biol.

[80] Liu C, Rokavec M, Huang Z, Hermeking H (2023). Curcumin activates a ROS/KEAP1/NRF2/miR-34a/b/c cascade to suppress colorectal cancer metastasis. Cell Death Differ.

[81] Zhang Z, Chen Q, Huang C, Rao D, Sang C, Zhu S (2022). Transcription factor Nrf2 binds to circRNAPIBF1 to regulate SOD2 in lung adenocarcinoma progression. Mol Carcinog.

[82] Fan JB, Zhang Y, Liu W, Zhu XH, Xu DW, Zhao JN (2018). Long Non-Coding RNA MALAT1 Protects Human Osteoblasts from Dexamethasone-Induced Injury via Activation of PPM1E-AMPK Signaling. Cell Physiol Biochem.

[83] Gai C, Liu C, Wu X, Yu M, Zheng J, Zhang W (2020). MT1DP loaded by folate-modified liposomes sensitizes erastin-induced ferroptosis via regulating miR-365a-3p/NRF2 axis in non-small cell lung cancer cells. Cell Death Dis.

[84] Kang KA, Piao MJ, Kim KC, Kang HK, Chang WY, Park IC (2014). Epigenetic modification of Nrf2 in 5-fluorouracil-resistant colon cancer cells: involvement of TET-dependent DNA demethylation. Cell Death Dis.

[85] Kang KA, Piao MJ, Hyun YJ, Zhen AX, Cho SJ, Ahn MJ (2019). Luteolin promotes apoptotic cell death via upregulation of Nrf2 expression by DNA demethylase and the interaction of Nrf2 with p53 in human colon cancer cells. Exp Mol Med.

[86] Fang X, Lee YH, Jang JH, Kim SJ, Kim SH, Kim DH (2023). ARD1 stabilizes NRF2 through direct interaction and promotes colon cancer progression. Life Sci.

[87] Guo H, Xu J, Zheng Q, He J, Zhou W, Wang K (2019). NRF2 SUMOylation promotes de novo serine synthesis and maintains HCC tumorigenesis. Cancer Lett.

[88] Sanghvi VR, Leibold J, Mina M, Mohan P, Berishaj M, Li Z (2019). The Oncogenic Action of NRF2 Depends on De-glycation by Fructosamine-3-Kinase. Cell.

[89] Tran KT, Pallesen JS, Solbak SMO, Narayanan D, Baig A, Zang J (2019). A Comparative Assessment Study of Known Small-Molecule Keap1-Nrf2 Protein-Protein Interaction Inhibitors: Chemical Synthesis, Binding Properties, and Cellular Activity. J Med Chem.

[90] Esteras N, Abramov AY (2022). Nrf2 as a regulator of mitochondrial function: Energy metabolism and beyond. Free Radic Biol Med.

[91] Wang R, Liang L, Matsumoto M, Iwata K, Umemura A, He F (2023). Reactive Oxygen Species and NRF2 Signaling, Friends or Foes in Cancer?. Biomolecules.

[92] Yamamoto M, Kensler TW, Motohashi H (2018). The KEAP1-NRF2 System: a Thiol-Based Sensor-Effector Apparatus for Maintaining Redox Homeostasis. Physiol Rev.

[93] Lu MC, Ji JA, Jiang ZY, You QD (2016). The Keap1-Nrf2-ARE Pathway As a Potential Preventive and Therapeutic Target: An Update. Med Res Rev.

[94] Wei R, Zhao Y, Wang J, Yang X, Li S, Wang Y (2021). Tagitinin C induces ferroptosis through PERK-Nrf2-HO-1 signaling pathway in colorectal cancer cells. Int J Biol Sci.

[95] Wu A, Feng B, Yu J, Yan L, Che L, Zhuo Y (2021). Fibroblast growth factor 21 attenuates iron overload-induced liver injury and fibrosis by inhibiting ferroptosis. Redox Biol.

[96] Meinert M, Jessen C, Hufnagel A, Kress JKC, Burnworth M, Daubler T (2024). Thiol starvation triggers melanoma state switching in an ATF4 and NRF2-dependent manner. Redox Biol.

[97] Rojo de la Vega M, Chapman E, Zhang DD (2018). NRF2 and the Hallmarks of Cancer. Cancer Cell.

[98] Li D, Shao R, Wang N, Zhou N, Du K, Shi J (2021). Sulforaphane Activates a lysosome-dependent transcriptional program to mitigate oxidative stress. Autophagy.

[99] Seok JH, Kim DH, Kim HJ, Jo HH, Kim EY, Jeong JH (2022). Epigallocatechin-3-gallate suppresses hemin-aggravated colon carcinogenesis through Nrf2-inhibited mitochondrial reactive oxygen species accumulation. J Vet Sci.

[100] Wang S, Wu YY, Wang X, Shen P, Jia Q, Yu S (2020). Lycopene prevents carcinogen-induced cutaneous tumor by enhancing activation of the Nrf2 pathway through p62-triggered autophagic Keap1 degradation. Aging (Albany NY).

[101] Zhou X, Zhao Y, Wang J, Wang X, Chen C, Yin D (2018). Resveratrol represses estrogen-induced mammary carcinogenesis through NRF2-UGT1A8-estrogen metabolic axis activation. Biochem Pharmacol.

[102] Wu TY, Saw CL, Khor TO, Pung D, Boyanapalli SS, Kong AN (2012). In vivo pharmacodynamics of indole-3-carbinol in the inhibition of prostate cancer in transgenic adenocarcinoma of mouse prostate (TRAMP) mice: involvement of Nrf2 and cell cycle/apoptosis signaling pathways. Mol Carcinog.

[103] Probst BL, McCauley L, Trevino I, Wigley WC, Ferguson DA (2015). Cancer Cell Growth Is Differentially Affected by Constitutive Activation of NRF2 by KEAP1 Deletion and Pharmacological Activation of NRF2 by the Synthetic Triterpenoid, RTA 405. PLoS One.

[104] Poornashree M, Kumar H, Ajmeer R, Jain R, Jain V (2023). Dual role of Nrf2 in cancer: molecular mechanisms, cellular functions and therapeutic interventions. Mol Biol Rep.

[105] Lee HR, Cho JM, Shin DH, Yong CS, Choi HG, Wakabayashi N (2008). Adaptive response to GSH depletion and resistance to L-buthionine-(S,R)-sulfoximine: involvement of Nrf2 activation. Mol Cell Biochem.

[106] Lu W, Cui J, Wang W, Hu Q, Xue Y, Liu X (2024). PPIA dictates NRF2 stability to promote lung cancer progression. Nat Commun.

[107] DeNicola GM, Karreth FA, Humpton TJ, Gopinathan A, Wei C, Frese K (2011). Oncogene-induced Nrf2 transcription promotes ROS detoxification and tumorigenesis. Nature.

[108] Hamad SH, Montgomery SA, Simon JM, Bowman BM, Spainhower KB, Murphy RM (2022). TP53, CDKN2A/P16, and NFE2L2/NRF2 regulate the incidence of pure- and combined-small cell lung cancer in mice. Oncogene.

[109] Lee HM, Muhammad N, Lieu EL, Cai F, Mu J, Ha YS (2024). Concurrent loss of LKB1 and KEAP1 enhances SHMT-mediated antioxidant defence in KRAS-mutant lung cancer. Nat Metab.

[110] Pillai R, LeBoeuf SE, Hao Y, New C, Blum JLE, Rashidfarrokhi A (2024). Glutamine antagonist DRP-104 suppresses tumor growth and enhances response to checkpoint blockade in KEAP1 mutant lung cancer. Sci Adv.

[111] Nioi P, Nguyen T (2007). A mutation of Keap1 found in breast cancer impairs its ability to repress Nrf2 activity. Biochem Biophys Res Commun.

[112] Kim YJ, Ahn JY, Liang P, Ip C, Zhang Y, Park YM (2007). Human prx1 gene is a target of Nrf2 and is up-regulated by hypoxia/reoxygenation: implication to tumor biology. Cancer Res.

[113] Xu T, Yang Y, Chen Z, Wang J, Wang X, Zheng Y (2023). TNFAIP2 confers cisplatin resistance in head and neck squamous cell carcinoma via KEAP1/NRF2 signaling. J Exp Clin Cancer Res.

[114] Rawat L, Balan M, Sasamoto Y, Sabarwal A, Pal S (2023). A novel combination therapy with Cabozantinib and Honokiol effectively inhibits c-Met-Nrf2-induced renal tumor growth through increased oxidative stress. Redox Biol.

[115] Lu Z, Ren Y, Yang L, Jia A, Hu Y, Zhao Y (2021). Inhibiting autophagy enhances sulforaphane-induced apoptosis via targeting NRF2 in esophageal squamous cell carcinoma. Acta Pharm Sin B.

[116] Hsu WL, Wang CM, Yao CL, Chen SC, Nien CY, Sun YH (2022). Blockage of Nrf2 and autophagy by L-selenocystine induces selective death in Nrf2-addicted colorectal cancer cells through p62-Keap-1-Nrf2 axis. Cell Death Dis.

[117] Lee Y, Chou TF, Pittman SK, Keith AL, Razani B, Weihl CC (2017). Keap1/Cullin3 Modulates p62/SQSTM1 Activity via UBA Domain Ubiquitination. Cell Rep.

[118] Schnupf P, Portnoy DA, Decatur AL (2006). Phosphorylation, ubiquitination and degradation of listeriolysin O in mammalian cells: role of the PEST-like sequence. Cell Microbiol.

[119] Zhang W, Geng X, Dong Q, Li X, Ye P, Lin M (2023). Crosstalk between autophagy and the Keap1-Nrf2-ARE pathway regulates realgar-induced neurotoxicity. J Ethnopharmacol.

[120] Jain A, Lamark T, Sjottem E, Larsen KB, Awuh JA, Overvatn A (2010). p62/SQSTM1 is a target gene for transcription factor NRF2 and creates a positive feedback loop by inducing antioxidant response element-driven gene transcription. J Biol Chem.

[121] Xu F, Xie Q, Li YW, Jing QQ, Liu XJ, Xu YC (2022). Suppression of JNK/ERK dependent autophagy enhances Jaspine B derivative-induced gastric cancer cell death via attenuation of p62/Keap1/Nrf2 pathways. Toxicol Appl Pharmacol.

[122] Liu JZ, Hu YL, Feng Y, Jiang Y, Guo YB, Liu YF (2020). BDH2 triggers ROS-induced cell death and autophagy by promoting Nrf2 ubiquitination in gastric cancer. J Exp Clin Cancer Res.

[123] Xie C, Zhou X, Liang C, Li X, Ge M, Chen Y (2021). Correction to: Apatinib triggers autophagic and apoptotic cell death via VEGFR2/STAT3/PD-L1 and ROS/Nrf2/p62 signaling in lung cancer. J Exp Clin Cancer Res.

[124] Murata H, Takamatsu H, Liu S, Kataoka K, Huh NH, Sakaguchi M (2015). NRF2 Regulates PINK1 Expression under Oxidative Stress Conditions. PLoS One.

[125] Gumeni S, Papanagnou ED, Manola MS, Trougakos IP (2021). Nrf2 activation induces mitophagy and reverses Parkin/Pink1 knock down-mediated neuronal and muscle degeneration phenotypes. Cell Death Dis.

[126] Moore TM, Cheng L, Wolf DM, Ngo J, Segawa M, Zhu X (2022). Parkin regulates adiposity by coordinating mitophagy with mitochondrial biogenesis in white adipocytes. Nat Commun.

[127] Yamada T, Dawson TM, Yanagawa T, Iijima M, Sesaki H (2019). SQSTM1/p62 promotes mitochondrial ubiquitination independently of PINK1 and PRKN/parkin in mitophagy. Autophagy.

[128] Heo JM, Ordureau A, Paulo JA, Rinehart J, Harper JW (2015). The PINK1-PARKIN Mitochondrial Ubiquitylation Pathway Drives a Program of OPTN/NDP52 Recruitment and TBK1 Activation to Promote Mitophagy. Mol Cell.

[129] Sun X, Ou Z, Chen R, Niu X, Chen D, Kang R (2016). Activation of the p62-Keap1-NRF2 pathway protects against ferroptosis in hepatocellular carcinoma cells. Hepatology.

[130] Liu P, Anandhan A, Chen J, Shakya A, Dodson M, Ooi A (2023). Decreased autophagosome biogenesis, reduced NRF2, and enhanced ferroptotic cell death are underlying molecular mechanisms of non-alcoholic fatty liver disease. Redox Biol.

[131] Singh A, Misra V, Thimmulappa RK, Lee H, Ames S, Hoque MO (2006). Dysfunctional KEAP1-NRF2 interaction in non-small-cell lung cancer. PLoS Med.

[132] Komatsu M, Kurokawa H, Waguri S, Taguchi K, Kobayashi A, Ichimura Y (2010). The selective autophagy substrate p62 activates the stress responsive transcription factor Nrf2 through inactivation of Keap1. Nat Cell Biol.

[133] Inami Y, Waguri S, Sakamoto A, Kouno T, Nakada K, Hino O (2011). Persistent activation of Nrf2 through p62 in hepatocellular carcinoma cells. J Cell Biol.

[134] Ichimura Y, Waguri S, Sou YS, Kageyama S, Hasegawa J, Ishimura R (2013). Phosphorylation of p62 activates the Keap1-Nrf2 pathway during selective autophagy. Mol Cell.

[135] Ren X, Li Y, Zhou Y, Hu W, Yang C, Jing Q (2021). Overcoming the compensatory elevation of NRF2 renders hepatocellular carcinoma cells more vulnerable to disulfiram/copper-induced ferroptosis. Redox Biol.

[136] Wang J, Liu Z, Hu T, Han L, Yu S, Yao Y (2017). Nrf2 promotes progression of non-small cell lung cancer through activating autophagy. Cell Cycle.

[137] Shankar S, Ganapathy S, Srivastava RK (2008). Sulforaphane enhances the therapeutic potential of TRAIL in prostate cancer orthotopic model through regulation of apoptosis, metastasis, and angiogenesis. Clin Cancer Res.

[138] Darvekar SR, Elvenes J, Brenne HB, Johansen T, Sjottem E (2014). SPBP is a sulforaphane induced transcriptional coactivator of NRF2 regulating expression of the autophagy receptor p62/SQSTM1. PLoS One.

[139] Jin CY, Yu HY, Park C, Han MH, Hong SH, Kim KS (2013). Oleifolioside B-mediated autophagy promotes apoptosis in A549 human non-small cell lung cancer cells. Int J Oncol.

[140] Wang Y, Zhang J, Huang ZH, Huang XH, Zheng WB, Yin XF (2017). Isodeoxyelephantopin induces protective autophagy in lung cancer cells via Nrf2-p62-keap1 feedback loop. Cell Death Dis.

[141] Xie C, Zhou X, Liang C, Li X, Ge M, Chen Y (2021). Apatinib triggers autophagic and apoptotic cell death via VEGFR2/STAT3/PD-L1 and ROS/Nrf2/p62 signaling in lung cancer. J Exp Clin Cancer Res.

[142] Yu H, Wu CL, Wang X, Ban Q, Quan C, Liu M (2019). SP600125 enhances C-2-induced cell death by the switch from autophagy to apoptosis in bladder cancer cells. J Exp Clin Cancer Res.

[143] Sun SY, Rosenberg LM, Wang X, Zhou Z, Yue P, Fu H (2005). Activation of Akt and eIF4E survival pathways by rapamycin-mediated mammalian target of rapamycin inhibition. Cancer Res.

[144] Li S, Yang G, Zhu X, Cheng L, Sun Y, Zhao Z (2017). Combination of rapamycin and garlic-derived S-allylmercaptocysteine induces colon cancer cell apoptosis and suppresses tumor growth in xenograft nude mice through autophagy/p62/Nrf2 pathway. Oncol Rep.

[145] Tuttis K, Machado ART, Santos P, Antunes LMG (2023). Sulforaphane Combined with Vitamin D Induces Cytotoxicity Mediated by Oxidative Stress, DNA Damage, Autophagy, and JNK/MAPK Pathway Modulation in Human Prostate Tumor Cells. Nutrients.

[146] Xia H, Zhang H, Ruan Z, Zhang H, Sun L, Chen H (2024). Neoadjuvant camrelizumab (an anti-PD-1 antibody) plus chemotherapy or apatinib (a VEGFR-2 inhibitor) for initially unresectable stage II-III non-small-cell lung cancer: a multicentre, two-arm, phase 2 exploratory study. Signal Transduct Target Ther.

[147] Sun X, Li J, Li Y, Wang S, Li Q (2020). Apatinib, a Novel Tyrosine Kinase Inhibitor, Promotes ROS-Dependent Apoptosis and Autophagy via the Nrf2/HO-1 Pathway in Ovarian Cancer Cells. Oxid Med Cell Longev.

[148] Schaff LR, Mellinghoff IK (2023). Glioblastoma and Other Primary Brain Malignancies in Adults: A Review. Jama-J Am Med Assoc.

[149] Mao LL, Lian B, Li CL, Bai X, Zhou L, Cui CL (2023). Camrelizumab Plus Apatinib and Temozolomide as First-Line Treatment in Patients With Advanced Acral Melanoma The CAP 03 Phase 2 Nonrandomized Clinical Trial. Jama Oncol.

[150] Zhou Y, Wang HD, Zhu L, Cong ZX, Li N, Ji XJ (2013). Knockdown of Nrf2 enhances autophagy induced by temozolomide in U251 human glioma cell line. Oncol Rep.

[151] THORPE JN (1951). Procaine with hyaluronidase as local anesthetic. Lancet.

[152] Bhol CS, Mishra SR, Patil S, Sahu SK, Kirtana R, Manna S (2022). PAX9 reactivation by inhibiting DNA methyltransferase triggers antitumor effect in oral squamous cell carcinoma. Biochim Biophys Acta Mol Basis Dis.

[153] Chen Y, Shi J, Wang X, Zhou L, Wang Q, Xie Y (2023). An antioxidant feedforward cycle coordinated by linker histone variant H1.2 and NRF2 that drives nonsmall cell lung cancer progression. Proc Natl Acad Sci U S A.

[154] Lister A, Nedjadi T, Kitteringham NR, Campbell F, Costello E, Lloyd B (2011). Nrf2 is overexpressed in pancreatic cancer: implications for cell proliferation and therapy. Mol Cancer.

[155] Zhang M, Hong X, Ma N, Wei Z, Ci X, Zhang S (2023). The promoting effect and mechanism of Nrf2 on cell metastasis in cervical cancer. J Transl Med.

[156] Wolowczyk C, Neckmann U, Aure MR, Hall M, Johannessen B, Zhao S (2022). NRF2 drives an oxidative stress response predictive of breast cancer. Free Radic Biol Med.

[157] Luo RZ, Yuan ZY, Li M, Xi SY, Fu J, He J (2013). Accumulation of p62 is associated with poor prognosis in patients with triple-negative breast cancer. Onco Targets Ther.

[158] Inoue D, Suzuki T, Mitsuishi Y, Miki Y, Suzuki S, Sugawara S (2012). Accumulation of p62/SQSTM1 is associated with poor prognosis in patients with lung adenocarcinoma. Cancer Sci.

[159] Li H, Li J, Zhang G, Da Q, Chen L, Yu S (2019). HMGB1-Induced p62 Overexpression Promotes Snail-Mediated Epithelial-Mesenchymal Transition in Glioblastoma Cells via the Degradation of GSK-3beta. Theranostics.

[160] Xi SY, Lu JB, Chen JW, Cao Y, Luo RZ, Wu QL (2013). The "stone-like" pattern of LC3A expression and its clinicopathologic significance in hepatocellular carcinoma. Biochem Biophys Res Commun.

[161] Wu WY, Kim H, Zhang CL, Meng XL, Wu ZS (2014). Clinical significance of autophagic protein LC3 levels and its correlation with XIAP expression in hepatocellular carcinoma. Med Oncol.

[162] Shi M, An G, Chen N, Jia J, Cui X, Zhan T (2023). UVRAG Promotes Tumor Progression through Regulating SP1 in Colorectal Cancer. Cancers (Basel).

[163] Feng X, Jia Y, Zhang Y, Ma F, Zhu Y, Hong X (2019). Ubiquitination of UVRAG by SMURF1 promotes autophagosome maturation and inhibits hepatocellular carcinoma growth. Autophagy.

[164] Wu DH, Wang TT, Ruan DY, Li X, Chen ZH, Wen JY (2018). Combination of ULK1 and LC3B improve prognosis assessment of hepatocellular carcinoma. Biomed Pharmacother.

[165] Tang J, Deng R, Luo RZ, Shen GP, Cai MY, Du ZM (2012). Low expression of ULK1 is associated with operable breast cancer progression and is an adverse prognostic marker of survival for patients. Breast Cancer Res Treat.

[166] Yun M, Bai HY, Zhang JX, Rong J, Weng HW, Zheng ZS (2015). ULK1: a promising biomarker in predicting poor prognosis and therapeutic response in human nasopharygeal carcinoma. PLoS One.

[167] Du H, Chen L, Luo F, Chen X, Li Y, Cheng Q (2020). Beclin-1 expression is associated with prognosis in a Bcl-2-dependent manner in non-small cell lung cancer. Oncol Lett.

[168] Zheng HC, Zhao S, Xue H, Zhao EH, Jiang HM, Hao CL (2020). The Roles of Beclin 1 Expression in Gastric Cancer: A Marker for Carcinogenesis, Aggressive Behaviors and Favorable Prognosis, and a Target of Gene Therapy. Front Oncol.

[169] Dong LW, Hou YJ, Tan YX, Tang L, Pan YF, Wang M (2011). Prognostic significance of Beclin 1 in intrahepatic cholangiocellular carcinoma. Autophagy.

[170] Paik PK, Fan PD, Qeriqi B, Namakydoust A, Daly B, Ahn L (2023). Targeting NFE2L2/KEAP1 Mutations in Advanced NSCLC With the TORC1/2 Inhibitor TAK-228. J Thorac Oncol.

[171] Silvis MR, Silva D, Rohweder R, Schuman S, Gudipaty S, Truong A (2023). MYC-mediated resistance to trametinib and HCQ in PDAC is overcome by CDK4/6 and lysosomal inhibition. J Exp Med.

[172] Pan J, Zhang M, Dong L, Ji S, Zhang J, Zhang S (2023). Genome-Scale CRISPR screen identifies LAPTM5 driving lenvatinib resistance in hepatocellular carcinoma. Autophagy.

[173] Zhou Y, Chen Y, Shi Y, Wu L, Tan Y, Li T (2023). FAM117B promotes gastric cancer growth and drug resistance by targeting the KEAP1/NRF2 signaling pathway. J Clin Invest.

[174] Cheung EC, Vousden KH (2022). The role of ROS in tumour development and progression. Nat Rev Cancer.

[175] Chen P, Zhong X, Song Y, Zhong W, Wang S, Wang J (2024). Triptolide induces apoptosis and cytoprotective autophagy by ROS accumulation via directly targeting peroxiredoxin 2 in gastric cancer cells. Cancer Lett.

[176] Yamamoto K, Venida A, Yano J, Biancur DE, Kakiuchi M, Gupta S (2020). Autophagy promotes immune evasion of pancreatic cancer by degrading MHC-I. Nature.

[177] Mukhopadhyay S, Mahapatra KK, Praharaj PP, Patil S, Bhutia SK (2022). Recent progress of autophagy signaling in tumor microenvironment and its targeting for possible cancer therapeutics. Semin Cancer Biol.

[178] Chen N, Debnath J (2013). IkappaB kinase complex (IKK) triggers detachment-induced autophagy in mammary epithelial cells independently of the PI3K-AKT-MTORC1 pathway. Autophagy.

[179] Di Martino JS, Nobre AR, Mondal C, Taha I, Farias EF, Fertig EJ (2022). A tumor-derived type III collagen-rich ECM niche regulates tumor cell dormancy. Nat Cancer.

[180] Massague J, Ganesh K (2021). Metastasis-Initiating Cells and Ecosystems. Cancer Discov.

[181] Pohl C, Dikic I (2019). Cellular quality control by the ubiquitin-proteasome system and autophagy. Science.

[182] Laval T, Ouimet M (2023). A role for lipophagy in atherosclerosis. Nat Rev Cardiol.

[183] Tang J, Peng W, Ji J, Peng C, Wang T, Yang P (2023). GPR176 Promotes Cancer Progression by Interacting with G Protein GNAS to Restrain Cell Mitophagy in Colorectal Cancer. Adv Sci (Weinh).

[184] Zhao M, Wang Y, Li L, Liu S, Wang C, Yuan Y (2021). Mitochondrial ROS promote mitochondrial dysfunction and inflammation in ischemic acute kidney injury by disrupting TFAM-mediated mtDNA maintenance. Theranostics.

[185] Deretic V, Saitoh T, Akira S (2013). Autophagy in infection, inflammation and immunity. Nat Rev Immunol.

[186] Jiang B, Cui Y, Ma X, Zhang Y, Feng X, Yang T (2023). Crosstalk between autophagy inhibitor and salidroside-induced apoptosis: A novel strategy for autophagy-based treatment of hepatocellular cancer. Int Immunopharmacol.

[187] Park SH, Kim JH, Chi GY, Kim GY, Chang YC, Moon SK (2012). Induction of apoptosis and autophagy by sodium selenite in A549 human lung carcinoma cells through generation of reactive oxygen species. Toxicol Lett.

[188] Pandey A, Trigun SK (2023). Fisetin induces apoptosis in colorectal cancer cells by suppressing autophagy and down-regulating nuclear factor erythroid 2-related factor 2 (Nrf2). J Cell Biochem.

[189] Huber S, Valente S, Chaimbault P, Schohn H (2014). Evaluation of ∆2-pioglitazone, an analogue of pioglitazone, on colon cancer cell survival: Evidence of drug treatment association with autophagy and activation of the Nrf2/Keap1 pathway. Int J Oncol.

[190] Sena P, Mancini S, Benincasa M, Mariani F, Palumbo C, Roncucci L (2018). Metformin Induces Apoptosis and Alters Cellular Responses to Oxidative Stress in Ht29 Colon Cancer Cells: Preliminary Findings. Int J Mol Sci.

[191] Neitzel C, Seiwert N, Goder A, Diehl E, Weber C, Nagel G (2019). Lipoic Acid Synergizes with Antineoplastic Drugs in Colorectal Cancer by Targeting p53 for Proteasomal Degradation. Cells.

[192] Celesia A, Morana O, Fiore T, Pellerito C, D'Anneo A, Lauricella M (2020). ROS-Dependent ER Stress and Autophagy Mediate the Anti-Tumor Effects of Tributyltin (IV) Ferulate in Colon Cancer Cells. Int J Mol Sci.

[193] Enkhbat T, Nishi M, Yoshikawa K, Jun H, Tokunaga T, Takasu C (2018). Epigallocatechin-3-gallate Enhances Radiation Sensitivity in Colorectal Cancer Cells Through Nrf2 Activation and Autophagy. Anticancer Res.

[194] Panigrahi DP, Bhol CS, R N, Nagini S, Patil S, Maiti TK (2020). Abrus agglutinin inhibits oral carcinogenesis through inactivation of NRF2 signaling pathway. Int J Biol Macromol.

[195] Wu Z, Su M, Chen H, Chen X, Chen CY, An L (2023). Sinularin Exerts Anti-cancer Effects by Inducing Oxidative Stress-mediated Ferroptosis, Apoptosis, and Autophagy in Prostate Cancer Cells. Anticancer Agents Med Chem.

[196] Xiong H, Chen Z, Lin B, Xie B, Liu X, Chen C (2021). Naringenin Regulates FKBP4/NR3C1/NRF2 Axis in Autophagy and Proliferation of Breast Cancer and Differentiation and Maturation of Dendritic Cell. Front Immunol.

[197] Long FY, Wang PH, Ma Y, Zhang XD, Wang T (2024). Chemopreventive effects of atractylenolide-III on mammary tumorigenesis via activation of the Nrf2/ARE pathway through autophagic degradation of Keap1. Biomedicine & Pharmacotherapy.

[198] Gonzalez Y, Aryal B, Chehab L, Rao VA (2014). Atg7- and Keap1-dependent autophagy protects breast cancer cell lines against mitoquinone-induced oxidative stress. Oncotarget.

[199] Wu CF, Lee MG, El-Shazly M, Lai KH, Ke SC, Su CW (2018). Isoaaptamine Induces T-47D Cells Apoptosis and Autophagy via Oxidative Stress. Mar Drugs.

[200] Xiao S, Liu N, Yang X, Ji G, Li M (2021). Polygalacin D suppresses esophageal squamous cell carcinoma growth and metastasis through regulating miR-142-5p/Nrf2 axis. Free Radic Biol Med.

[201] Shin D, Kim EH, Lee J, Roh JL (2017). RITA plus 3-MA overcomes chemoresistance of head and neck cancer cells via dual inhibition of autophagy and antioxidant systems. Redox Biol.

[202] Lae Lae Phoo N, Sukhamwang A, Dejkriengkraikul P, Yodkeeree S (2022). Diclofenac Sensitizes Signet Ring Cell Gastric Carcinoma Cells to Cisplatin by Activating Autophagy and Inhibition of Survival Signal Pathways. Int J Mol Sci.

[203] Gilardini Montani MS, Cecere N, Granato M, Romeo MA, Falcinelli L, Ciciarelli U (2019). Mutant p53, Stabilized by Its Interplay with HSP90, Activates a Positive Feed-Back Loop Between NRF2 and p62 that Induces Chemo-Resistance to Apigenin in Pancreatic Cancer Cells. Cancers (Basel).

[204] Cheng L, Yan B, Chen K, Jiang Z, Zhou C, Cao J (2018). Resveratrol-Induced Downregulation of NAF-1 Enhances the Sensitivity of Pancreatic Cancer Cells to Gemcitabine via the ROS/Nrf2 Signaling Pathways. Oxid Med Cell Longev.

[205] Xiu Z, Zhu Y, Han J, Li Y, Yang X, Yang G (2022). Caryophyllene Oxide Induces Ferritinophagy by Regulating the NCOA4/FTH1/LC3 Pathway in Hepatocellular Carcinoma. Front Pharmacol.

[206] Jiang Z, Gao L, Liu C, Wang J, Han Y, Pan J (2023). Sarmentosin Induces Autophagy-dependent Apoptosis via Activation of Nrf2 in Hepatocellular Carcinoma. J Clin Transl Hepatol.

[207] Zhang L, Wang H (2017). FTY720 inhibits the Nrf2/ARE pathway in human glioblastoma cell lines and sensitizes glioblastoma cells to temozolomide. Pharmacol Rep.

[208] Jang JE, Eom JI, Jeung HK, Chung H, Kim YR, Kim JS (2020). PERK/NRF2 and autophagy form a resistance mechanism against G9a inhibition in leukemia stem cells. J Exp Clin Cancer Res.

[209] Du Z, Wang Q, Ma G, Jiao J, Jiang D, Zheng X (2019). Inhibition of Nrf2 promotes the antitumor effect of Pinelliae rhizome in papillary thyroid cancer. J Cell Physiol.

[210] Merikallio H, Paakko P, Kinnula VL, Harju T, Soini Y (2012). Nuclear factor erythroid-derived 2-like 2 (Nrf2) and DJ1 are prognostic factors in lung cancer. Hum Pathol.

[211] Xia Q, Xie J, Zhang J, Zhang L, Zhou Y, Zhu B (2024). Ovatodiolide induces autophagy-mediated cell death through the p62-Keap1-Nrf2 signaling pathway in chronic myeloid leukemia cells. Chem Biol Interact.

[212] Jia X, He Q, Zeng M, Chen Y, Liu Y (2021). Activation of MEK1/2/Nrf-2 Signaling Pathway by Epstein-Barr Virus-Latent Membrane Protein 1 Enhances Autophagy and Cisplatin Resistance in T-Cell Lymphoma. Anal Cell Pathol (Amst).

[213] Yang F, Yan Z, Nie W, Liu Z, Cheng X, Wang W (2021). LACTB and LC3 could serve as potential biomarkers of gastric cancer to neoadjuvant chemotherapy with oxaliplatin plus S-1. Oncol Lett.

[214] Hara K, Horikoshi Y, Morimoto M, Nakaso K, Sunaguchi T, Kurashiki T (2023). TYRO3 promotes chemoresistance via increased LC3 expression in pancreatic cancer. Transl Oncol.

[215] Liu Y, Wang D, Lei M, Gao J, Cui Y, Jin X (2021). GABARAP suppresses EMT and breast cancer progression via the AKT/mTOR signaling pathway. Aging (Albany NY).

[216] Gil J, Ramsey D, Pawlowski P, Szmida E, Leszczynski P, Bebenek M (2018). The Influence of Tumor Microenvironment on ATG4D Gene Expression in Colorectal Cancer Patients. Med Oncol.

[217] Brigger D, Torbett BE, Chen J, Fey MF, Tschan MP (2013). Inhibition of GATE-16 attenuates ATRA-induced neutrophil differentiation of APL cells and interferes with autophagosome formation. Biochem Biophys Res Commun.

[218] Roberts SS, Mendonca-Torres MC, Jensen K, Francis GL, Vasko V (2009). GABA receptor expression in benign and malignant thyroid tumors. Pathol Oncol Res.

